# PPM1M, an LRRK2-counteracting, phosphoRab12-preferring phosphatase with a potential link to Parkinson’s disease

**DOI:** 10.1016/j.celrep.2025.116031

**Published:** 2025-07-20

**Authors:** Claire Y. Chiang, Neringa Pratuseviciute, Yu-En Lin, Ayan Adhikari, Wondwossen M. Yeshaw, Chloe Flitton, Pemba L. Sherpa, Francesca Tonelli, Irena Rektorova, Timothy Lynch, Joanna Siuda, Monika Rudzińska-Bar, Oleksandr Pulyk, Peter Bauer, Christian Beetz, Dennis W. Dickson, Owen A. Ross, Zbigniew K. Wszolek, Global Parkinson’s Genetics Program (GP2), Zih-Hua Fang, Christine Klein, Alexander Zimprich, Dario R. Alessi, Esther M. Sammler, Suzanne R. Pfeffer

**Affiliations:** 1 Department of Biochemistry, Stanford University School of Medicine, Stanford, CA, USA; 2 Aligning Science Across Parkinson’s (ASAP) Collaborative Research Network, Chevy Chase, MD, USA; 3 MRC Protein Phosphorylation and Ubiquitylation Unit, University of Dundee, Dundee, UK; 4 School of Medicine and St Anne’s Hospital, 1st Department of Neurology, Pekarska 53, Brno, Czech Republic; 5 Department of Neurology, Dublin Neurological Institute, Dublin, Ireland; 6 Śląski Uniwersytet Medyczny w Katowicach, Katowice, Poland; 7 Department of Neurology, Andrzej Frycz Modrzewski University, Krakow, Poland; 8 Wladyslaw Bieganski Collegium Medicum Jan Długosz University, Częstochowa, Poland; 9 CENTOGENE GmbH, Rostock, Germany; 10 Mayo Clinic Florida, Department of Neuroscience, Jacksonville, FL 32224, USA; 11 German Center for Neurodegenerative Diseases, DZNE, Tübingen, Germany; 12 Institute of Neurogenetics, University of Lübeck, Lübeck, Germany; 13 Department of Neurology, Medical University of Vienna, Vienna, Austria; 14 Lead contact

## Abstract

Leucine-rich repeat kinase 2 (LRRK2) phosphorylates a subset of Rab GTPases that regulate receptor trafficking, and *LRRK2*-activating mutations are linked to Parkinson’s disease. Rab phosphorylation is a transient event that can be reversed by phosphatases, including protein phosphatase, Mg2^+^/Mn2^+^ dependent 1H (PPM1H), which acts on phosphorylated Rab 8A (phosphoRab8A) and phosphoRab10. Here, we report a phosphatome-wide small interfering RNA (siRNA) screen that identified PPM1M as a phosphoRab12-preferring phosphatase that also acts on phosphoRab8A and phosphoRab10. Upon knockout from cultured cells or mice, PPM1M displays selectivity for phosphoRab12. As shown previously for mice harboring LRRK2 pathway mutations, knockout of *Ppm1m* leads to primary cilia loss in striatal cholinergic and parvalbumin interneurons. We also identified a rare *PPM1M* mutation in patients with Parkinson’s disease that is catalytically inactive when tested *in vitro* and in cells. These findings identify PPM1M as a key player in the LRRK2 signaling pathway and provide a new therapeutic target for the possible benefit of patients with Parkinson’s disease.

## In brief

Leucine-rich repeat kinase 2 (LRRK2) phosphorylates a subset of Rab GTPases that regulate receptor trafficking; *LRRK2*-activating mutations are linked to Parkinson’s disease. Chiang et al. reveal that PPM1M reverses Rab phosphorylation *in vitro* and *in vivo*, and a rare inactivating mutation is ∼6-fold enriched in people with Parkinson’s disease.

## Graphical Abstract

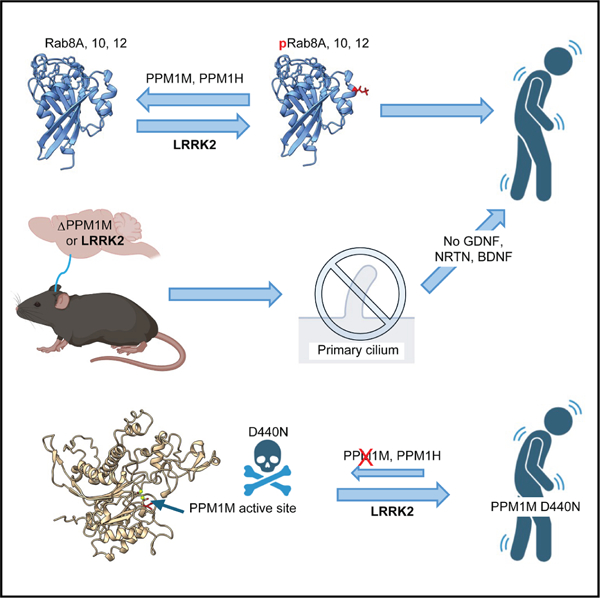

## INTRODUCTION

While the cause of most Parkinson’s disease (PD) cases is unknown, perhaps as many as 15% of PD cases are due to genetic variation.^1–4^ Of these, around 3% are due to activating variants in the leucine-rich repeat kinase 2 (*LRRK2*) gene,^3–5^ making it one of the most clinically relevant PD genes. *LRRK2* pathogenic variants display population-specific frequencies.^2,6^ The most common global pathogenic *LRRK2* variant is G2019S, which increases kinase activity about 2-fold.^7^ *LRRK2* G2019S has a high frequency in Ashkenazi Jewish (15%–20%) and North African Arab-Berber (>30%) patients with PD.^2,6^ Other population-specific variants include *LRRK2* R1441G (Basque population, ∼6%)^2,6^ and R1628P and G2385R (East Asian population, 5%–10% of patients; 2-fold risk increase). Notably, a protective *LRRK2* haplotype (N551K-R1398H-K1423K) has also been identified, suggesting that kinase activity—and therefore disease risk—is modifiable.^6,8^ LRRK2 is considered a highly promising target for PD treatment, and several clinical trials are underway.^9^ Unlike current PD symptomatic treatments, LRRK2-specific kinase inhibitors could potentially slow or halt PD progression or might even prevent disease manifestation.^10^ Moreover, LRRK2 inhibitors would likely benefit patients with both *LRRK2* variant and idiopathic forms of PD, as elevated LRRK2 activity has been reported in such cases.^11^

LRRK2 phosphorylates a subset of the 65 human Rab GTPases^12,13^ that are master regulators of membrane trafficking.^14,15^ Upon phosphorylation, these Rab GTPases (hereafter referred to as phosphoRabs) bind to a new set of phosphoRab-specific effector proteins to drive cellular pathology.^13^ In 2019, Berndsen et al.^16^ reported the discovery of PPM1H (protein phosphatase, Mg2^+^/Mn2^+^ dependent 1H) as a Rab GTPase-specific, LRRK2 action-reversing phosphatase. PPM1H shows a preference for Rab8A, Rab10, and Rab35, and dephosphorylation of these Rabs regulates primary cilia formation^16^ and autophagy.^17^ PPM1H relies on an N-terminal amphipathic helix for Golgi localization in cells,^18^ and under certain conditions, it can be detected on mitochondrial surfaces.^19^ PPM1H is most active when associated with highly curved membranes, where the interaction of its N-terminal amphipathic helix with those membranes enhances its catalytic activity.^18^ PPM1H’s specificity for LRRK2-phosphorylated Rab proteins is largely determined by a unique structural feature called a “flap” domain.^20^ Domain-swap experiments have shown that this 110-residue domain, located adjacent to the active site, has evolved to recognize specific phosphoRabs.^20^ PPM1H’s ability to reverse pathological Rab phosphorylation positions it as another potential therapeutic target for PD, with activity enhancers offering a novel approach to modulate LRRK2 signaling and mitigate disease progression.

While PPM1H is highly specific for certain Rab proteins, it does not act efficiently on all LRRK2-phosphorylated Rabs. For example, Rab12 is a poor substrate for PPM1H in cellular contexts,^16^ suggesting that other phosphatases may also play a role. Thus, we carried out two additional, small interfering RNA (siRNA)-based, phosphatome-wide screens to identify additional Rab-specific phosphatases. We present here the discovery of the PPM1H-related PPM1M as a phosphoRab12-preferring, Rab-specific phosphatase that has a rare, PD-associated, catalytically inactivating mutation.

## RESULTS

### PPM1H does not act alone: Evidence for additional Rab phosphatases

**Figure 1.**
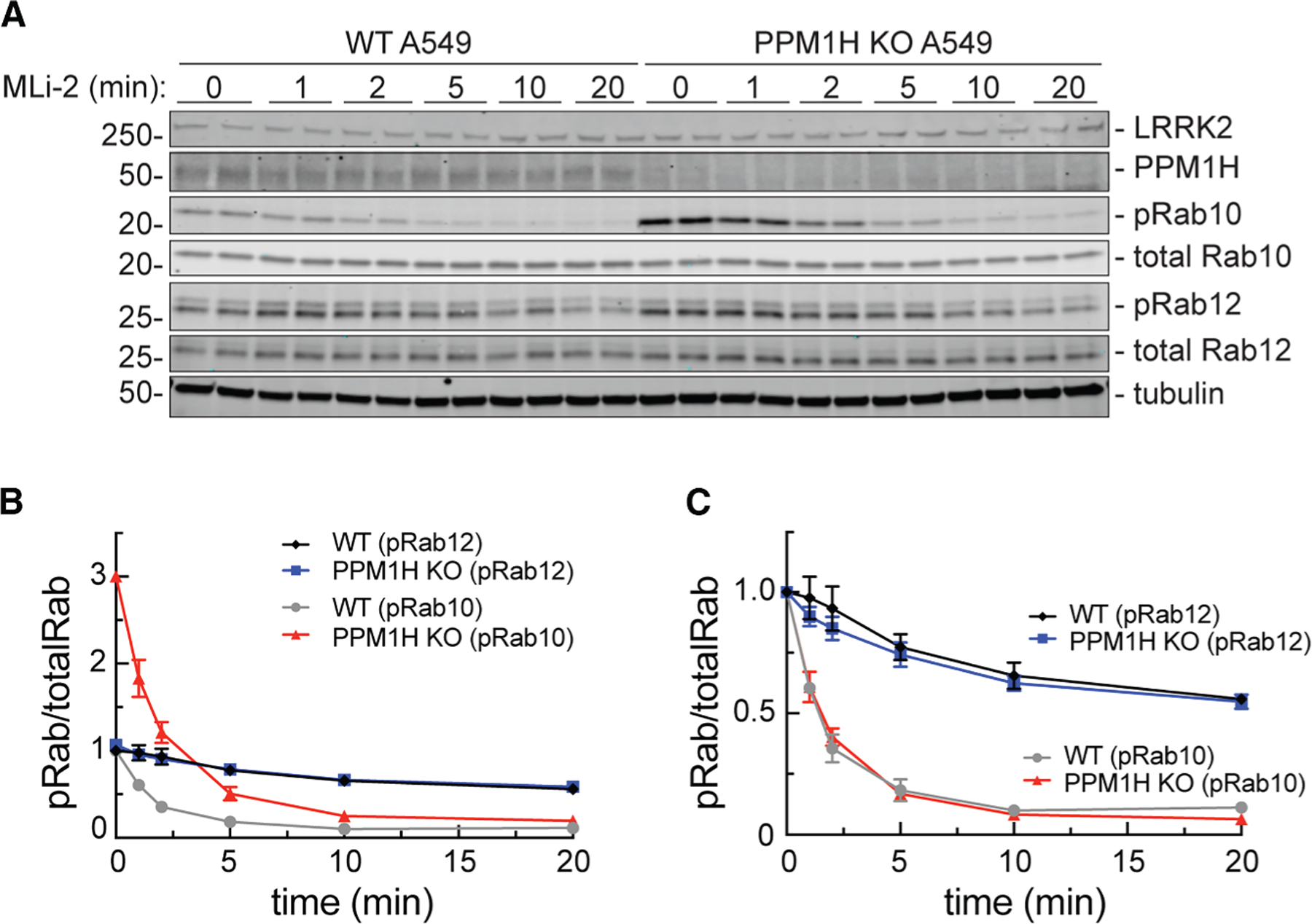
PPM1H knockout does not influence the rate of phosphoRab turnover (A) Immunoblot analysis of wild-type (WT) and PPM1H knockout (KO) A549 cells treated with 100 nM MLi-2 for the times indicated. Mass is shown on the left in kDa here and in all subsequent figures; antigens are indicated on the right. (B) Quantitation of phosphoRab10 (pRab10)/total Rab10 and phosphoRab12 (pRab12)/total Rab12 levels from immunoblots in (A), normalized to 1.0 for respective pRab10 or pRab12 WT 0 min conditions, as indicated. (C) Quantitation of pRab10/total Rab10 and pRab12/total Rab12 levels from immunoblots in (A), normalized to 1.0 for 0 min of each respective (WT or KO) condition to permit direct comparison. Error bars represent SEM from 3 independent experiments carried out in duplicate.

Detailed analysis revealed that PPM1H activity is not sufficient to explain phosphoRab10 dephosphorylation kinetics. To measure PPM1H-dependent dephosphorylation, we compared phosphoRab levels in cells with and without PPM1H protein; the LRRK2 kinase inhibitor MLi-2^21^ was added for various lengths of time to block Rab protein re-phosphorylation (Figure 1A). At baseline, phosphoRab10 levels were 3-fold higher in PPM1H knockout (KO) cells relative to wild-type (WT) cells (Figure 1B, red versus gray symbols), while phosphoRab12 levels were unchanged (Figure 1B, blue and black symbols). Thus, as we have reported previously,^16^ phosphoRab12 levels are not impacted by the loss of PPM1H.

Normalization of the data for direct comparison revealed that both phosphoRab10 and phosphoRab12 continue to be dephosphorylated at equal rates, irrespective of the presence of PPM1H (Figure 1C). Under these conditions, phosphoRab10 turned over very quickly, with a half-life of about 2 min, while phosphoRab12 displayed a half-life of 20 min. These data demonstrate the existence of another phosphoRab10 phosphatase(s), at least in A549 cells, with a preference for phosphoRab10 over phosphoRab12. A549 cells were used in these experiments as they had been previously used to identify PPM1H^16^ and have good levels of endogenous LRRK2.

### Phosphatome-wide screen identifies PPM1M as another phosphoRab phosphatase

**Figure 2.**
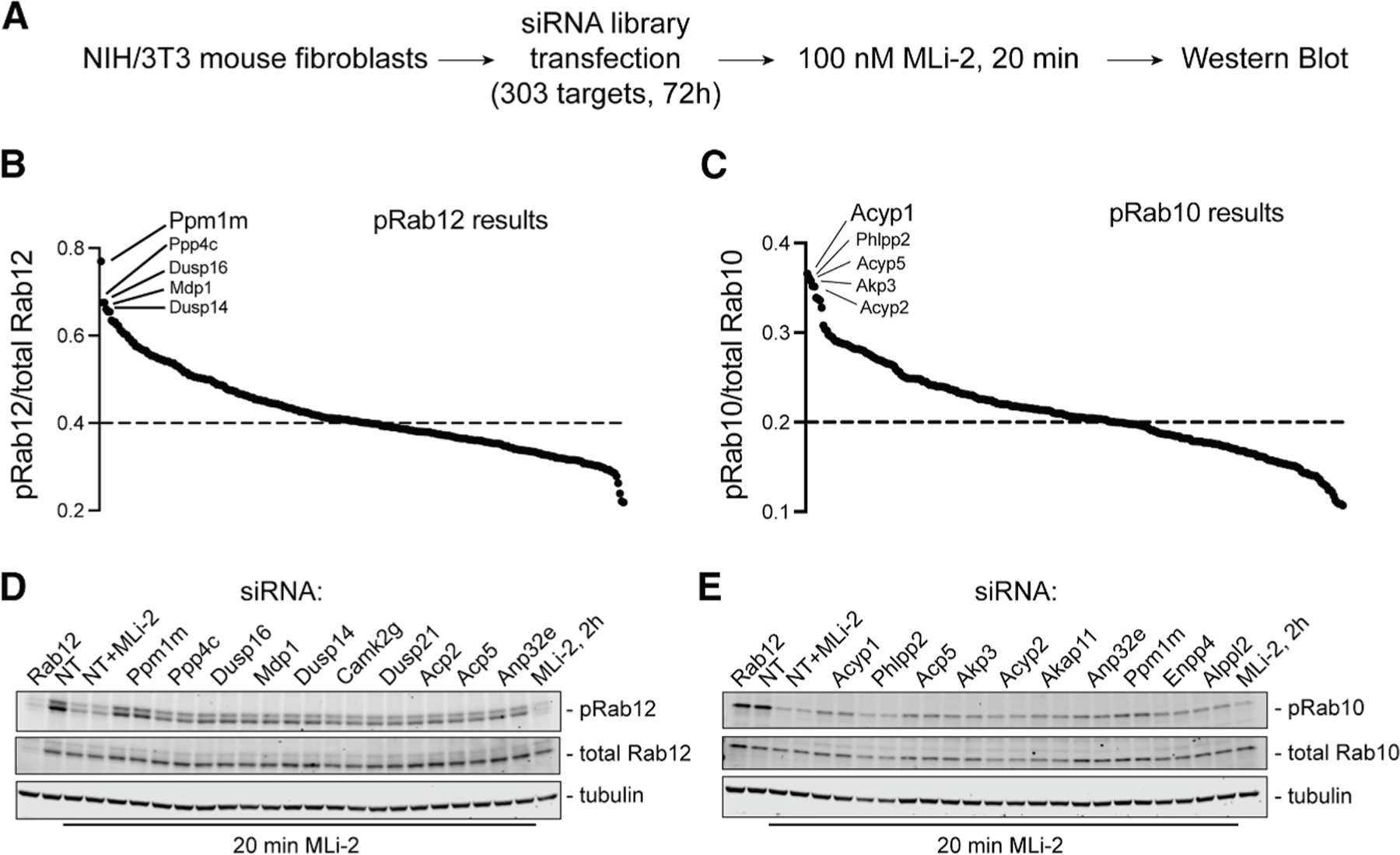
Phosphatome-wide siRNA screen in 3T3 cells reveals PPM1M as a phosphoRab12-preferring phosphatase (A) Schematic describing screen workflow. (B and C) Summary plot of (B) pRab12/total Rab12 or (C) pRab10/total Rab10 levels after 72 h siRNA and 20 min MLi-2 treatment, normalized to non-targeting (NT) control without MLi-2 treatment. The top 5 hits for each pRab are indicated. (D and E) Repeat immunoblots of the lysates of the top 10 hits from (B) for pRab12 and from (C) for pRab10. The NT+MLi-2 condition is treatment with 100 nM MLi-2 for 20 min, and the MLi-2, 2h condition is treatment with 100 nM MLi-2 for 2 h.

We performed a phosphatome-wide siRNA screen to identify a novel phosphoRab12 phosphatase and/or a second phosphoRab10 phosphatase. Our screen compared 303 siRNAs targeting phosphatases and corresponding regulatory subunits in mouse 3T3 cells (Figure 2A; Table S1). 3T3 cells were selected because they have performed well in prior CRISPR screens,^22^ and, importantly, they contain sufficient LRRK2 to enable detection of endogenous phosphoRab10 and phosphoRab12. To identify an LRRK2-counteracting phosphoRab12 phosphatase, we chose a longer duration of MLi-2 treatment (20 min) than used previously (5 min for phosphoRab10 in Berndsen et al.^16^) to best evaluate phosphoRab12 turnover, as phosphoRab12 turns over more slowly than phosphoRab10 (Figure 1). After 72 h with siRNA, cells were treated with MLi-2 (100 nM, 20 min) and then analyzed by immunoblot for phosphoRab10 and phosphoRab12 levels (Figures 2, S1, and S2).

Figures 2B and 2C summarize the results of the screen, monitoring phosphoRab12 (Figure 2B) and phosphoRab10 (Figure 2C) levels, normalized to a non-targeting (NT) siRNA control. Genes that, when knocked down, yielded the highest levels of a phosphoRab were considered the most significant LRRK2-counteracting phosphatases. siRNA samples for the top 10 hits for each phosphoRab were re-analyzed to validate our findings (Figures 2D and 2E); note that, as before, phosphoRab levels are monitored after 20 min of MLi-2 treatment, in comparison with the maximum signal seen in the NT control without MLi-2 treatment. PPM1M was the top hit for phosphoRab12, and it was also among the top 10 hits for phosphoRab10. PPM1M is very closely related to PPM1H (∼45% identity) and is a member of the PPM1H subfamily of PPM phosphatases, along with PPM1J.^23^ Note that PPM1H was not identified in this screen to be a top hit for phosphoRab10, likely due to its absence or extremely low expression in the 3T3 cells used here; the prior phosphoRab10 phosphatase screen was carried out in A549 cells.^16^

As expected, siRNA knockdown of Rab12 decreased total Rab12 but not total Rab10 levels. siRNA knockdown of Rab12 also decreased phosphoRab10 levels (normalized to total Rab10) because Rab12 activates LRRK2 Rab10 phosphorylation.^22,24^ In additional controls, NT siRNA samples treated with 100 nM MLi-2 for 2 h also showed significant decreases in both phosphoRab10 and phosphoRab12 levels due to LRRK2 inhibition, confirming the activity of MLi-2. PPM1M knockdown in mouse embryonic fibroblasts (MEFs) also yielded an increase in phosphoRab12 levels, validating PPM1M’s role in a different cell line (Figures S3A and S3B).

### PPM1M overexpression influences phosphoRab12 and phosphoRab10 levels

**Figure 3.**
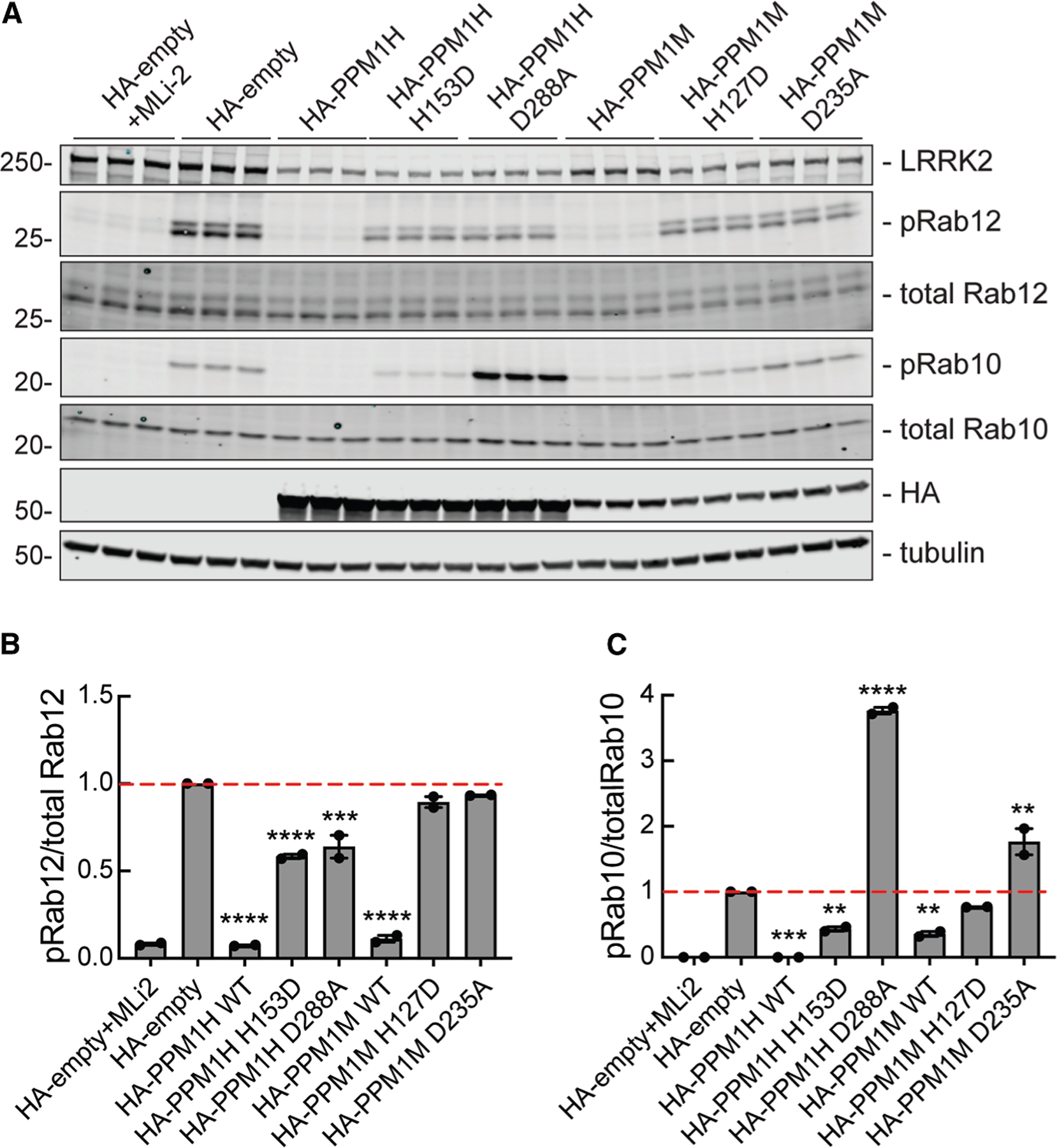
PPM1M overexpression preferentially decreases phosphoRab12 compared with phosphoRab10 (A) Immunoblot analysis of HEK293 cells over-expressing FLAG-LRRK2 R1441C and HA-empty or HA-tagged PPM1H, PPM1H H153D, PPM1H D288A, PPM1M, PPM1M H127D, or PPM1M D235A. Cells were treated with 200 nM MLi-2 for 2 h where indicated. (B and C) Quantitation of (B) pRab12/total Rab12 and (C) pRab10/total Rab10 levels from immunoblots in (A), normalized to 1.0 for HA-empty. Error bars indicate SEM from two independent experiments analyzed in duplicate. Statistical significance was determined by one-way ANOVA, relative to HA-empty. For pRab10, ***p = 0.0002 for PPM1H WT, **p = 0.0097 for PPM1H H153D, ****p < 0.0001 for PPM1H D288A, **p = 0.0044 for PPM1M WT, and **p = 0.0014 for PPM1M D235A. For pRab12, ****p < 0.0001 for PPM1H WT, PPM1H H153D, and PPM1M WT and ***p = 0.0002 for PPM1H D288A.

Exogenous expression of PPM1M and PPM1H was used to assess the effects of these enzymes on phosphoRab10 and phosphoRab12, along with LRRK2 R1441C in HEK293 cells. LRRK2 R1441C was used because this mutation provides the highest level of LRRK2 activity upon overexpression; HEK293 cells do not have much endogenous LRRK2. We also overexpressed PPM1H and PPM1M catalytically inactive mutants (PPM1H H153D and PPM1M H127D^16^) and substrate-trapping mutants (PPM1H D288A and PPM1M D235A^16^) in HEK293 cells, along with LRRK2 R1441C (Figure 3A). Overexpression experiments were performed in HEK293 cells because these cells enable efficient co-transfection of all components.

Note that hemagglutinin-epitope tagged (HA)-PPM1H is expressed ∼2-fold higher (lanes 7–15) than HA-PPM1M (lanes 16–24) after the transfection of cells with the same amounts of plasmid. LRRK2 expression also appears to be slightly higher when transfected together with HA-PPM1M compared with HA-PPM1H, likely due to plasmid competition in cells. Nevertheless, as expected, exogenously expressed PPM1H fully dephosphorylated phosphoRab10 and mostly dephosphorylated phosphoRab12 (Figure 3A, lanes 7–9). PPM1M almost completely dephosphorylated phosphoRab12 and mostly dephosphorylated phosphoRab10 (Figure 3A, lanes 16–18). Thus, upon over-expression, PPM1H and PPM1M can both dephosphorylate phosphoRab12, but PPM1H dephosphorylates phosphoRab10 more efficiently than PPM1M.

Also, as expected, catalytically inactive mutants PPM1H H153D and PPM1M H127D failed to dephosphorylate either phosphoRab substrate (Figure 3). The PPM1H D288A substrate-trapping mutant increased phosphoRab10 levels about 4-fold, as previously shown,^16^ likely by shielding phosphoRab10 from other phosphatases. The PPM1M D235A substrate-trapping mutant increased phosphoRab10 levels about 2-fold (Figure 3C). Neither substrate-trapping mutant increased phosphoRab12 levels, suggesting that phosphoRab12 may be unique in the way that it engages these phosphatases (Figure 3B). In summary, overexpression experiments show that PPM1M can work on both phosphoRab10 and phosphoRab12 substrates, with a preference for phosphoRab12.

### PPM1M KO confirms preference for phosphoRab12 in culture and in mouse tissue

**Figure 4.**
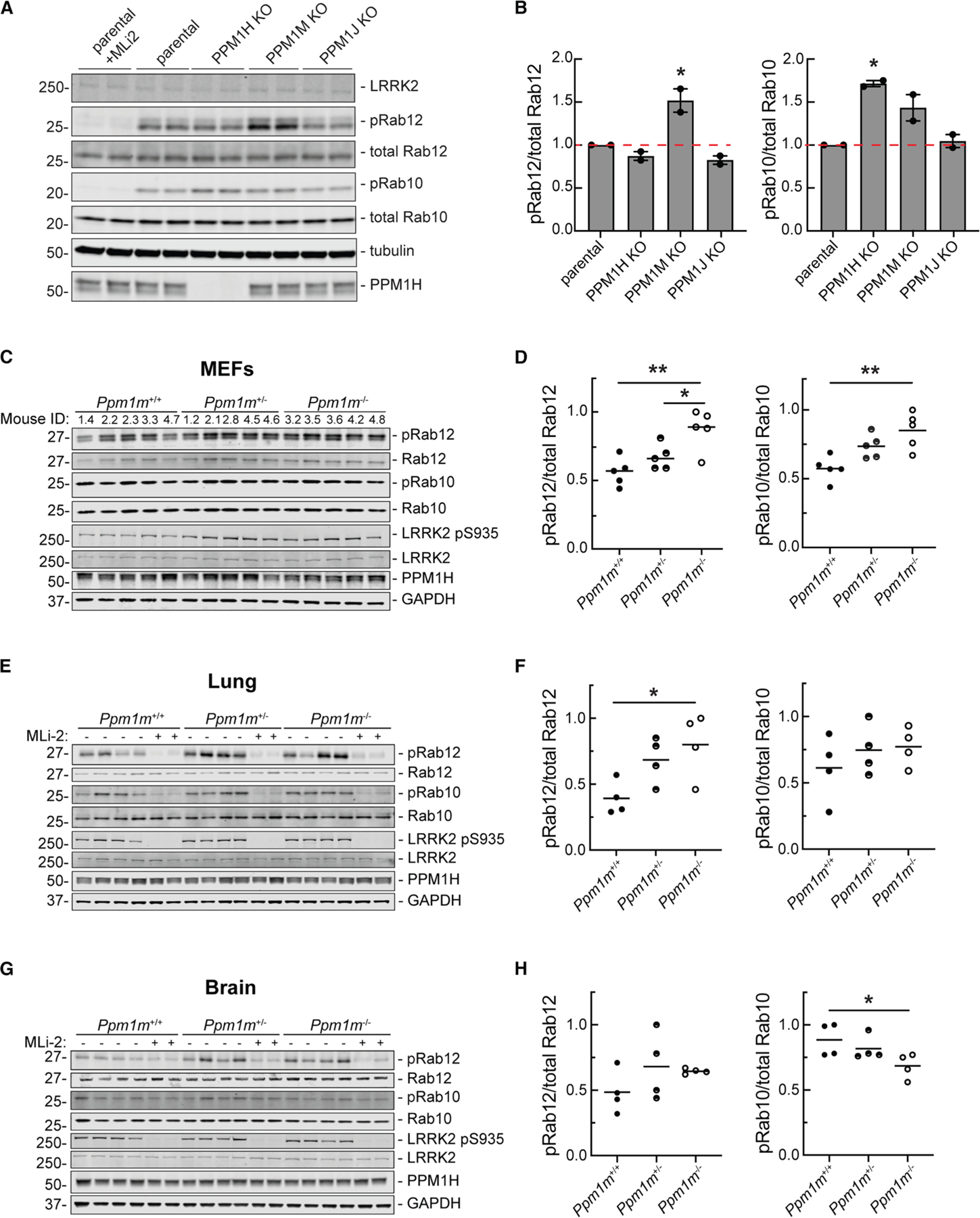
Knockout of PPM1 subfamily phosphatases in MEF cells and tissues confirms PPM1M substrate preferences (A) Immunoblot analysis of parental (wild-type) and PPM1H, PPM1M, and PPM1J pooled knockouts in MEF cells. (B) Quantitation of pRab12/total Rab12 and pRab10/total Rab10 levels from immunoblots in (A), normalized to 1.0 for parental. Error bars indicate SEM from two independent experiments analyzed in duplicate. Statistical significance was determined by one-way ANOVA, respective to parental. For pRab10, *p = 0.0122 for PPM1H knockout. For pRab12, *p = 0.0117 for PPM1M knockout. (C) Immunoblot analysis of mouse embryonic fibroblasts (MEFs) derived from *Ppm1m* wild-type (*Ppm1m*^+/+^), heterozygous knockout (*Ppm1m*^+/−^), or homozygous knockout (*Ppm1m*^−/−^) mice. (D) Quantitation of pRab12/total Rab12 and pRab10/total Rab10 levels from immunoblots in (C), normalized to 1.0 for the highest value. Each dot represents the average of two independent replicates from one mouse. Statistical significance was determined by one-way ANOVA. For pRab10, **p = 0.0036 and for pRab12, *p = 0.0399 and **p = 0.0029. (E) Immunoblot analysis of lung lysates from mice as in (C). (F) Quantitation of immunoblots in (E), normalized as in (D). Each dot represents the average of three independent replicates from one mouse. Statistical significance was determined by one-way ANOVA. For pRab12, *p = 0.0335. (G) Immunoblot analysis of whole-brain lysates from mice as in (C). (H) Quantitation from immunoblots in (G) as in (D). Statistical significance was determined by Kruskal-Wallis test for pRab10 and one-way ANOVA for pRab12. For pRab10, *p = 0.0349.

We generated pooled CRISPR KO MEF cells to obtain lines without *Ppm1h*, *Ppm1m*, or the related *Ppm1j* (Figure 4A). MEF cells were used here as they express reasonable levels of both PPM1H and PPM1M. Due to the unfortunate lack of a PPM1M or PPM1J antibody, CRISPR KO of the genes was confirmed by DNA sequencing (Figure S3C). Of the three phosphatases, only the absence of PPM1M increased phosphoRab12 levels (Figures 4A and 4B). For phosphoRab10, *Ppm1h* KO increased its levels by almost 2-fold, as reported previously^16^; *Ppm1m* KO also increased phosphoRab10 levels by about 50% (Figure 4B). *Ppm1j* KO did not influence either phosphoRab10 or phosphoRab12 (Figure 4B), and PPM1J has not shown activity on any phosphoRab tested to date (this study and Berndsen et al.^16^). It is possible that PPM1J is not expressed in these cells.

Similar experiments using MEF cells derived from homozygous *Ppm1m* KO mice showed increases in phosphoRab12 and phosphoRab10 levels (Figures 4C and 4D). Lung lysates from *Ppm1m* KO mice showed an increase in phosphoRab12 levels with no change in steady-state phosphoRab10 levels (Figures 4E and 4F). A similar trend of increased phosphoRab12 levels was observed in *Ppm1m* KO whole-brain lysates but did not reach significance (Figures 4G and 4H). The mouse KO data are consistent with the general preference of PPM1M for phosphoRab12 over phosphoRab10 in cultured cells and in tissues. The *Ppm1m* KO mouse exhibits no overt phenotype under standard husbandry conditions. Body weight, growth, and overall health appear normal, with no significant differences compared to WT littermates.

### Characterization of PPM1M

To further characterize PPM1M’s substrate preferences, we performed *in vitro* phosphatase assays using purified PPM1M and phosphoRab10 and phosphoRab12 (Figure S4A) in conjunction with phosphoRab-specific antibodies and immunoblotting to monitor phosphatase action. PPM1M displayed an approximately 2-fold preference for phosphoRab12 over phosphoRab10; dephosphorylation rates were measured at different enzyme concentrations (Figures S4C and S4D). We were not able to purify sufficient phosphoRab12 and active PPM1M to determine precise kcat/K_M_ values by colorimetric assays. Also note that, unlike PPM1H, the PPM1M enzyme lost significant activity upon freezing.

Phos-tag gels used to resolve phosphorylated and unphosphorylated Rab8A showed that PPM1M can also act on phosphoRab8A at similar ratios of enzyme to substrate, as employed in Figures S4C and S4D. In these experiments, PPM1M was as active as PPM1H in dephosphorylating Rab8A protein, while the catalytically inactive mutants (PPM1M H127D and PPM1H 153D) showed no activity (Figures S4E and S4F).

PPM1H is a dimer.^20^ When PPM1M was exogenously expressed in HEK293 cells and the cytosol fractionated by size-exclusion column chromatography, immunoblotting of column fractions indicated that PPM1M also exists as a dimer, based on its chromatographic properties in relation to marker proteins (Figure S4B).

### PPM1M flap domain contributes to its substrate selectivity

**Figure 5.**
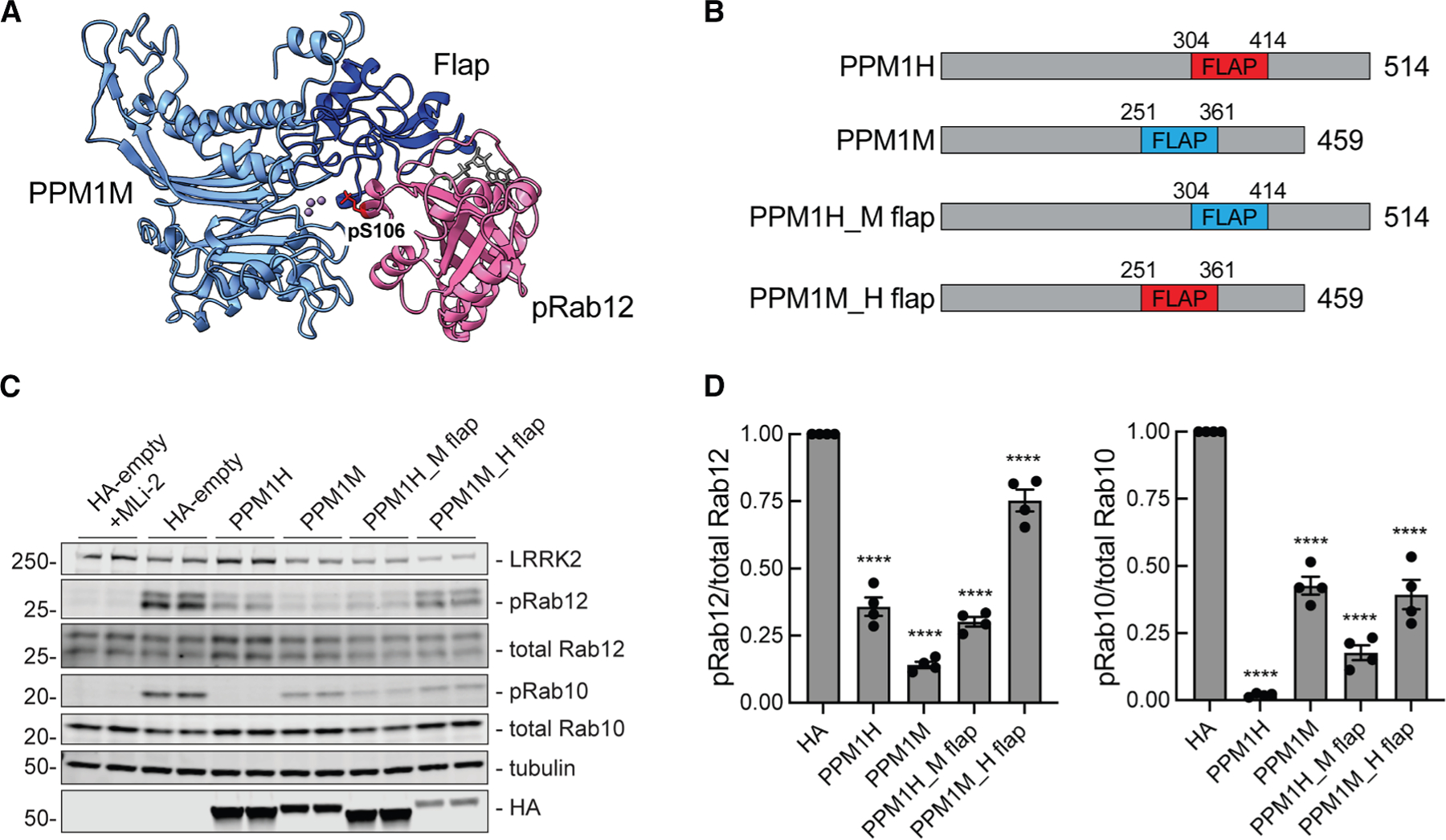
PPM1H and PPM1M flap domains are necessary for proper substrate recognition (A) AlphaFold modeling of PPM1M (blue) and pRab12 (magenta). The PPM1M flap domain is shown in navy; phosphoserine 106 of the pRab12 substrate is indicated at the metal-containing PPM1M active site. (B) Diagram of PPM1H and PPM1M swapped flap domain constructs. (C) Immunoblot analysis of HEK293 cells overexpressing FLAG-LRRK2 R1441C and HA-empty or HA-tagged PPM1H, PPM1M, PPM1H with PPM1M flap domain (PPM1H_M flap), or PPM1M with PPM1H flap domain (PPM1M_H flap). (D) Quantitation of pRab12/total Rab12 and pRab10/total Rab10 levels from immunoblots in (C), normalized to 1.0 for HA-empty. Error bars indicate SEM from four independent experiments analyzed in duplicate. Statistical significance was determined by one-way ANOVA, respective to HA-empty. ****p < 0.0001.

Elegant domain-swap experiments have shown that the flap domain, located adjacent to the enzyme active site and unique to PPM family phosphatases, is critical for substrate specificity (Figure 5A, navy blue^20^). Similar domain swaps between the flaps of PPM1H and PPM1M confirmed their roles in phosphoRab selectivity (Figures 5B–5D). Constructs were expressed in HEK293 cells in the presence of LRRK2 R1441C to compare phosphoRab12 and phosphoRab10 levels (Figures 5C and 5D). As expected, PPM1H preferentially dephosphorylated phosphoRab10 over phosphoRab12, while PPM1M dephosphorylated phosphoRab12 more than phosphoRab10. Compared to WT PPM1H, PPM1H containing the PPM1M flap domain (PPM1H_M flap) no longer dephosphorylated phosphoRab10 as efficiently, yet it retained a similar ability to work on phosphoRab12. PPM1M containing PPM1H’s flap domain (PPM1M_H flap) was expressed at about 2-fold lower levels than WT PPM1M. In this context, it was unable to dephosphorylate phosphoRab12 to the same extent as WT PPM1M despite dephosphorylating phosphoRab10 to a similar extent as WT PPM1M (Figure 5D). Therefore, PPM1H and PPM1M both likely rely on their flap domains for proper substrate recognition.

### *Ppm1m* KO phenocopies hyperactive *Lrrk2* knockin ciliation defect

**Figure 6.**
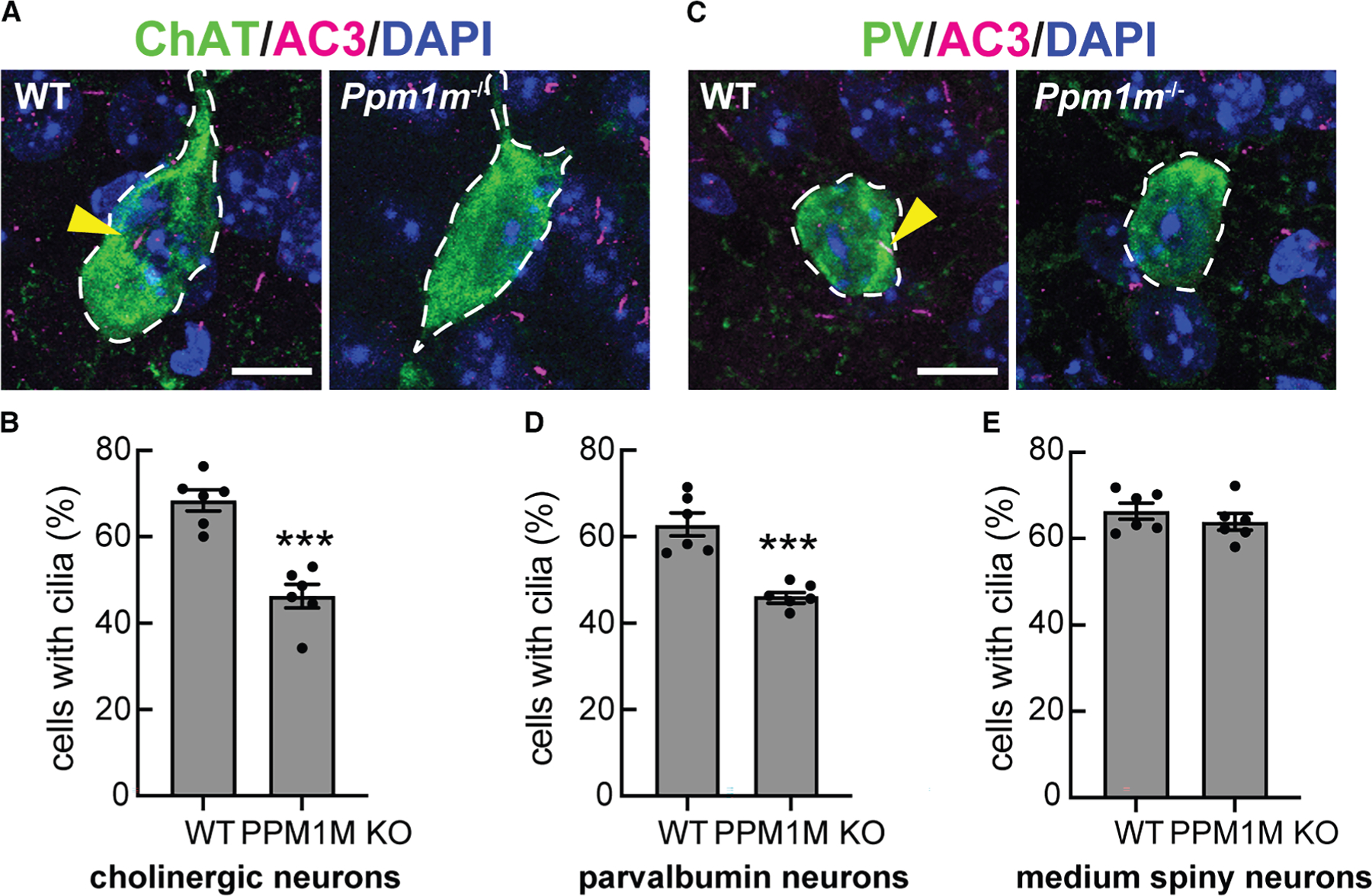
*Ppm1m* knockout phenocopies hyperactive *Lrrk2* ciliation phenotype (A) Example confocal immunofluorescence micrographs of sections of the dorsal striatum from 3-month-old wild-type or *Ppm1m*^−/−^ mice; scale bar, 10 μm. Cholinergic interneurons were labeled using anti-choline acetyltransferase (ChAT) antibody (green), and primary cilia were labeled using anti-AC3 (adenylate cyclase 3) antibody (magenta; yellow arrowhead). Nuclei were labeled using DAPI (blue). (B) Quantitation of ChAT^+^ neurons containing a cilium. (C) Example confocal immunofluorescence micrographs of sections of the dorsal striatum from 3-month-old wild-type or *Ppm1m*^−/−^ mice; scale bar, 10 μm. Parvalbumin neurons were labeled using anti-parvalbumin (PV) antibody (green); cilia and nuclei are labeled as in (A). (D) Quantitation of parvalbumin neurons containing a cilium. (E) Quantitation of surrounding, ChAT^−^ (mostly medium spiny) neurons containing a cilium. For (B), (D), and (E), error bars represent SEM from six individual brains per group, 2–3 sections per mouse. >36 ChAT^+^ neurons, >37 PV^+^ neurons, and >500 ChAT^−^ cells were scored per mouse. Statistical significance was determined using an unpaired t test. ***p = 0.0001 for cholinergic neurons, ***p = 0.0002 for parvalbumin neurons, and ns *p* = 0.3835 for medium spiny neurons.

We have shown previously that mice harboring hyperactive *Lrrk2* mutations (R1441C or G2019S) or lacking *Ppm1h* show loss of primary cilia in cholinergic and parvalbumin interneurons and astrocytes but not medium spiny neurons of the mouse dorsal striatum.^25–28^ Similar cilia losses are seen in the dorsal striatum from patients with idiopathic or LRRK2-pathway mutations.^27,28^ Therefore, we examined the ciliation status of cholinergic and parvalbumin interneurons of the dorsolateral striatum of mice lacking *Ppm1m*. As shown in Figure 6, while cholinergic interneurons were ∼70% ciliated in the dorsal striatum of WT mice, they were only ∼45% ciliated in *Ppm1m* KO mice (Figures 6A and 6B). Parvalbumin neurons were ∼65% ciliated in the dorsal striatum of WT mice and ∼45% ciliated in 3-month-old *Ppm1m* KO mice (Figures 6C and 6D). For comparison, *Ppm1h* knockout mice and *Lrrk2* G2019S knockin and bacterial artificial chromosome (BAC) transgene mice show similar extents of cilia loss (5-month-old PPM1H KO mouse decrease from ∼70% to ~35% in cholinergic neurons and from ~70% to ~45% in parvalbumin neurons^26^). As expected,^27,28^ there was no change in ciliation in the surrounding neurons, most of which are medium spiny neurons (Figure 6E). Thus, at least in cells of the dorsal striatum, PPM1M plays as significant a role in regulating the LRRK2 pathway as PPM1H or pathogenic LRRK2.

### *PPM1M* D440N mutant increases risk of PD

**Figure 7.**
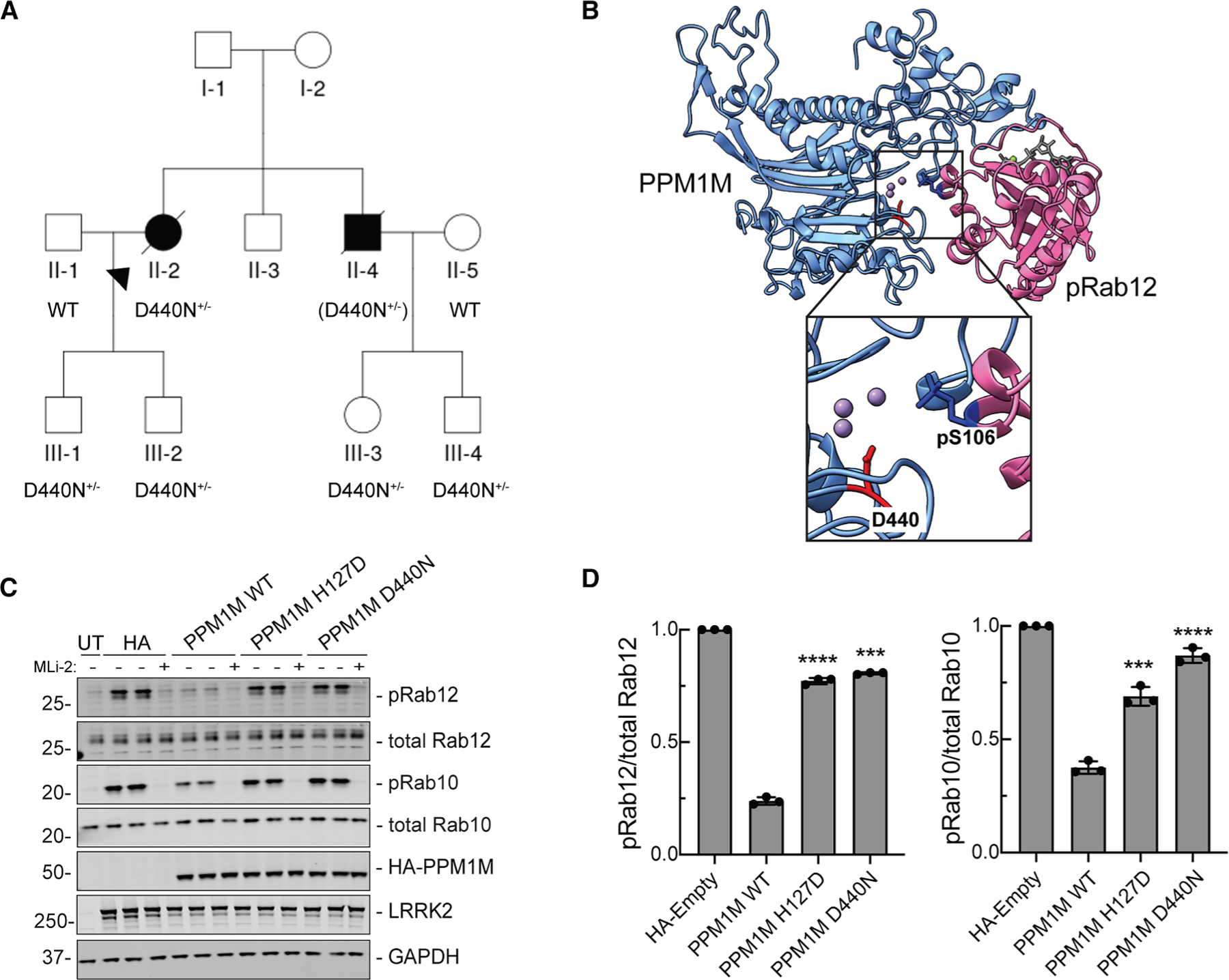
Patient with Parkinson’s disease linked to PPM1M D440N mutation (A) Individuals genotyped for the c.1318G>A p.D440N mutation are indicated as *D440N*^+/−^. In the case of II-4, the mutation was inferred from its presence in both of his children, III-3 and III-4, and is shown in parentheses (*D440N*^+/−^). A second-degree cousin of II-3 and II-4 also had PD but did not carry the D440N variant. The shared common ancestors of this individual with II-2 and II-4 are their great-grandparents (the grandparents of I-2, not shown in the pedigree). No clinical phenotype information for any of the members of generation III was available. (B) AlphaFold 3 modeling of D440 in the PPM1M (blue) active site shown with pRab12 (magenta, residues 38–222). Inset shows enlarged view of PPM1M D440 and Rab12 pS106. (C) Immunoblot analysis of HEK293 cells overexpressing FLAG-LRRK2 R1441G and HA-empty, HA-PPM1M WT, HA-PPM1M H127D, and HA-PPM1M D440N, with untransfected (UT) control. MLi-2 (200 nM) treatment was for 90 min as indicated. (D) Quantitation of pRab12/total Rab12 and pRab10/total Rab10 levels from immunoblots in (C), normalized to HA-empty. Error bars indicate SD from three independent experiments carried out in duplicate. Statistical significance was determined by Welch’s t test, followed by Benjamini-Hochberg correction for multiple comparisons, respective to HA-empty. ***p = 0.00011 for pRab12 - PPM1M D440N, ***p = 0.00078 for pRab10 - PPM1M H127D, and ****p < 0.0001 otherwise.

Mutations in *LRRK2* are identified as causal for PD, and a *PPM1H* truncating mutation has been identified in a PD case where, upon autopsy, it was found that the postmortem brain contained a cilia phenotype similar to that identified in LRRK2 mutation postmortem brains; *Ppm1h* KO in mouse shows the same phenotype as hyperactive *LRRK2* mutations.^27^ Therefore, we explored the possibility of a similar genetic link between *PPM1M* mutations and PD. Several recent studies identified *RAB32* p.R71R as a susceptibility variant for familial PD^29–31^ and also reported additional variants in other genes with strong associations with PD.^31^ Interestingly, among them was a rare missense variant in the *PPM1M* gene: NM_144641.4: c.1318G>A p.D440N (hereafter referred to as D440N). This variant was found in three out of 2,184 familial index patients with PD, with only three occurrences in a much larger control group of 69,775 subjects (odds ratio [OR]: 44, *p* = 3.81 × 10^−5^). For one of the three index patients, DNA was available for additional family members, allowing us to test for segregation of the *PPM1M* D440N variant (Figure 7A). The carrier status of the affected brother was inferred to be heterozygous for the *PPM1M* D440N variant, given that both children carried the variant. However, a second-degree cousin who also had PD did not carry the variant (Figure 7A).

We also identified the PPM1M D440N variant in 1 out of 382 patients from our Austrian cohort of individuals with PD (Table S2). Additionally, this variant was observed in seven other individuals from external cohorts with PD or PD-related disorders: once among 700 cases from the Mayo Clinic brain bank in a subject diagnosed with dementia with Lewy bodies; once in 725 PD cases from a Polish cohort; once in 139 Ukrainian PD cases; and in four individuals with PD or PD-related conditions from the Global Parkinson’s Genetics Program (GP2)—specifically, two with PD, one with progressive supranuclear palsy (PSP), and one with prodromal parkinsonism. However, we did not detect this variant in a cohort of 10,000 patients with PD from the CENTOGENE database, where exome or genome sequencing data were available (Table S2). In total, the PPM1M D440N variant was found in 11 of 28,167 individuals with PD or PD-related disorders, corresponding to a mutation frequency of 1.95 × 10^−4^. In control populations, the frequency was approximately six times lower (3.72 × 10^−5^), corresponding to an OR of 5.25 and a *p* value of 2.17 × 10^−5^. As described below, we have obtained additional functional evidence that *PPM1M* D440N may contribute to PD via indirect activation of the LRRK2 kinase pathway.

### PPM1M D440N is inactive

The *PPM1M* D440N mutation is located precisely in the middle of PPM1M’s active site (Figure 7B). Therefore, we tested if this mutation affects PPM1M phosphatase activity. Upon expression in HEK293 cells along with hyperactive LRRK2 R1441G, the PPM1M D440N mutant protein was inactive, comparable to the catalytically inactive PPM1M H127D mutation (Figures 7C and 7D). Similarly, *in vitro* dephosphorylation assays with purified proteins, visualized using Phos-tag gels, showed that PPM1M D440N is as inactive as PPM1M H127D on phosphoRab8A (Figure S4F). Future work will be needed to further characterize the possible contribution of the *PPM1M* D440N mutation to PD.

## DISCUSSION

We have shown here that, like PPM1H, the related PPM1M protein is a Rab phosphatase that counteracts LRRK2 phosphorylation. Although both phosphatases can act on phosphoRab8A and phosphoRab10, PPM1M is unique in its ability to act on phosphoRab12 at endogenous levels in 3T3 and MEF cells. In MEFs from a mouse *Ppm1m* KO model, we detect increased phosphoRab12 and phosphoRab10 levels. Similar results were obtained when comparing the relative consequences of *Ppm1m* and *Ppm1h* CRISPR KO in WT MEF cells. In lung lysates, we found that *Ppm1m* KO increases phosphoRab12 but not phosphoRab10 levels, with a similar trend in whole-brain lysates.

Using purified phosphatases and phosphorylated Rab proteins, PPM1M shows a roughly 2-fold preference for phosphoRab12 over phosphoRab10. Both PPM1H and PPM1M are expressed at low levels in most cells tested (approximately 10,000 copies per MEF cell), and the lack of available anti-PPM1M antibodies has made it challenging to study endogenous PPM1M. Nevertheless, both PPM1M’s and PPM1H’s roles as Rab phosphatases are now established through KO experiments in both cell culture and mouse models. Consistent with previous reports,^16^ we were unable to detect any activity for the related PPM1J protein. It is noteworthy that the KO of either PPM1H or PPM1M yields a ciliogenesis defect in cholinergic neurons of the dorsal striatum; this shows that the proteins act on the same pathway and do not seem to be sufficient to act in lieu of one another—double-KO mice will be needed to confirm this proposal.

AlphaFold modeling based on the crystal structure of PPM1H^20^ reveals that PPM1M shares a conserved phosphatase fold. The model also indicates that PPM1M has a similar overall structure, including a substrate-specifying flap domain. In general agreement with previously reported domain-swap experiments, the flap domain of PPM1H inserted in place of PPM1M’s flap domain decreases PPM1M’s ability to act on phosphoRab12. Similarly, insertion of the PPM1M flap into PPM1H slightly decreases its ability to dephosphorylate phosphoRab10. Although neither swap was perfect, these data support the conclusion that the flap domain is an important contributor to Rab substrate specificity of PPM family phosphatases.

PPM1H relies on an N-terminal amphipathic helix to bind highly curved liposomes *in vitro* and to localize approximately half of the protein to the Golgi in cultured cells.^18^ In contrast, PPM1M lacks this N-terminal amphipathic helix, consistent with our observation that exogenously expressed PPM1M appears entirely cytosolic by light microscopy of transfected 3T3 and A549 cells. Like PPM1H,^20^ PPM1M forms a dimer in the cytosol, as determined by gel filtration, and the recombinant protein maintains its dimeric state after purification from bacteria. Due to low PPM1M levels, we cannot rule out the possibility that a fraction of the endogenous protein is membrane associated; the protein may be transiently associated with membrane-associated substrate Rabs to enable dephosphorylation. We have obtained evidence for heterodimers when analyzing overexpressed PPM proteins, but these likely reflect a small proportion of the total enzyme pool.

PPM1H is widely expressed across various tissues, with the highest levels in the brain, whereas PPM1M is generally expressed at lower levels and is most abundant in neutrophils. Thus, the two proteins diverge in their expression patterns. Notably, while PPM1H is highly expressed in the brain, its levels vary significantly among different neuronal cell types. For example, in the striatum, we have shown that medium spiny neurons exhibit high LRRK2 and low PPM1H expression.^27^ Cholinergic interneurons, though, show the opposite pattern, with low LRRK2 and high PPM1H expression.^27^ Despite the apparently low expression in the brain, we detected increased levels of phosphoRab12 in brain extracts from PPM1M KO mice. PPM1M KO mice also show loss of primary cilia in cholinergic and parvalbumin neurons of the dorsal striatum, much like mice harboring hyperactive LRRK2- or PPM1H-inactivating mutations. The consequences of increased phosphoRab12 in the brains of these mice will be an important area for future investigation.

We identified a *PPM1M* variant that was reported to increase the risk of PD, D440N.^31^ Large-scale screening of available genetic data identified a number of patients with PD that carry the *PPM1M* D440N variant, and the occurrence of this variant is at least ∼6-fold higher in PD cohorts compared with extremely large control datasets. Determining the pathogenicity of rare variants is a growing challenge within the field of clinical genetics. While our extensive dataset provides statistically significant evidence of an association (OR = 5.25, *p* = 2.17 × 10^−5^), the absolute number of carriers is low, affecting roughly 1 in 2,500 individuals. This underscores the importance of caution before classifying this variant as definitively causing PD. However, we believe that functional assays, such as those presented here, will be essential in evaluating the pathogenic potential of rare genetic variants.

The PPM1M D440N mutation is located precisely in the phosphatase active site. Upon expression in cultured cells or after purification from bacteria, PPM1M D440N protein showed a complete loss of activity, supporting functional pathogenicity. Integration of functional data with further genetic screening efforts, at both the variant and gene levels, will help resolve the role of *PPM1M* variants in PD susceptibility.

### Limitations of the study

Despite the strong connection between PPM1H and PPM1M phosphatases and LRRK2 action, additional phosphatases are also clearly involved in regulating overall phosphoRab levels; this screen identified additional candidates, but more work is needed to identify the full repertoire of LRRK2-counteracting enzymes. Moreover, phosphoRab12 seems more resistant to phosphatases than phosphoRab10, based on dephosphorylation kinetics in A549, HEK293, 3T3, and MEF cells. Part of this resistance may be because Rab12 is also phosphorylated by additional kinases. Nevertheless, the screens presented herein and the prior Berndsen et al.^16^ screen have identified a group of additional phosphatases with the capacity to act on phosphorylated Rab proteins. Comparison of the top hits for phosphoRab12 and phosphoRab10 in this study and those of Berndsen et al.^16^ revealed essentially no overlap in other top hits, perhaps due to cell-type differences but also because the MLi-2 sensitization protocols used were distinct (20 min MLi-2 pretreatment herein versus 5 min in the prior study^16^). Additional phosphatases are especially relevant given the findings of Ito et al.,^32^ who have shown that Rab phosphorylation is a highly transient modification. Their data suggest that overall phosphorylation is regulated by a constant balance of kinase activation countered by dephosphorylation. Future experiments will provide additional insight into the link between LRRK2-dependent Rab phosphorylation and phosphatase action.

## RESOURCE AVAILABILITY

### Lead contact

Requests for further information and resources should be directed to and will be fulfilled by the lead contact, Suzanne R. Pfeffer (pfeffer@stanford.edu).

### Materials availability

All reagents used in this study are available from commercial sources or repositories without restriction, and the RRIDs for all reagents are provided in the key resources table.

## Data and code availability

- The data, protocols, and key lab materials used and generated in this study are listed in a key resources table alongside their persistent identifiers at Zenodo: https://doi.org/10.5281/zenodo.14911978 and https://doi.org/10.5281/zenodo.15499979. All data analysis and visualization were performed using GraphPad Prism. GP2’s Data Release 10 (Zenodo Data: https://doi.org/10.5281/zenodo.7904831) was used to screen for *PPM1M* p.D440N carriers. An earlier version of this manuscript was posted to bioRxiv on March 19, 2025, at https://doi.org/10.1101/2025.03.19.644182.
- This paper does not report original code.
- Any additional information required to re-analyze the data reported in this paper is available from the lead contact upon request.

## STAR★METHODS

### EXPERIMENTAL MODEL AND STUDY PARTICIPANT DETAILS

#### Cell culture

All commercial cell lines were validated by the vendor: HEK293 cells were purchased from ATCC; Flp-In 3T3 cells were purchased from Invitrogen (Carlsbad, CA). PPM1H knockout A549, wild-type MEF, and PPM1M knockout MEF cells were obtained from MRC-PPU. All cells were cultured in DMEM high-glucose media (Cytiva, Marlborough, MA) with 10% fetal bovine serum (Sigma, St. Louis, MO) and 1% Penicillin-Streptomycin (Sigma) and grown at 37°C, 5% CO_2_ in a humidified atmosphere and regularly tested for *Mycoplasma* contamination. See first section here for detailed cell culture protocol https://dx.doi.org/10.17504/protocols.io.kxygxyk9dl8j/v1.

#### Mice

Mice were generated at the Baylor College of Medicine as part of the Baylor College of Medicine, Sanger Institute, and MRC Harwell (BaSH) Consortium for the NIH Common Fund program for Knockout Mouse Production and Cryopreservation (1U42RR033192–01) and Knockout Mouse Phenotyping (1U54HG006348–01). Mice are distributed by MRC Harwell on behalf of MMRRC. More information can be found here: https://www.informatics.jax.org/allele/MGI:5638564. Ppm1m^tm2b(EUCOMM)Hmgu^ mice based on C57BL/6N-Atm1Brd (3 months old) were used. 7 female and 5 male mice were used for brain analyses; 11 males and 7 females were used for immunoblotting with 3 homozygous males and 3 homozygous females compared with 4 male and 2 wild type female mice. Gender did not appear to influence Rab phosphorylation or ciliogenesis in this study but larger cohorts would need to be analyzed to provide adequate power in support of this conclusion.

#### Research standards for animal studies

Mice were maintained under specific pathogen-free conditions at the University of Dundee (UK). All animal experiments were ethically reviewed and conducted in compliance with the Animals (Scientific Procedures) Act 1986 and guidelines established by the University of Dundee and the U.K. Home Office. Ethical approval for animal studies and breeding was obtained from the University of Dundee ethical committee, and all procedures were performed under a U.K. Home Office project license. The mice were group-housed in an environment with controlled ambient temperature (20°C–24°C) and humidity (45–55%), following a 12-h light/12-h dark cycle, with *ad libitum* access to food and water.

#### Genomic analysis

The sample sizes and clinical designations for the various volunteer cohorts are presented in Table S2.

### METHOD DETAILS

#### Cloning and plasmids

DNA constructs were amplified in *E. coli* DH5α and purified using mini prep columns (Econospin, Fisher Scientific). Whole plasmid DNA sequence verification was performed by Primordium/Plasmidsaurus (https://plasmidsaurus.com). pET15b His-MST3 was a kind gift of Amir Khan (Trinity College, Dublin). pCMV5D HA-empty or HA-PPM1H, PPM1H H153D, PPM1H D288A, PPM1M, PPM1M H127D, PPM1M D235A, and PPM1M D440N were obtained from MRC PPU. pET15b His-SUMO-Rab10 Q68L and Rab12 Q101L were previously obtained or cloned. pET15b His-SUMO-PPM1M was cloned using the pET15b backbone and HA-PPM1M insert via Gibson assembly. A detailed protocol for Gibson assembly can be found here https://doi.org/10.17504/protocols.io.eq2lyjwyqlx9/v1. See key resources table for access to plasmids from MRC PPU and/or Addgene.

#### Transient overexpression in HEK293 cells

For transient overexpression assays in HEK293 cells, cells were plated in 2 mL complete media in 6-well plates 16–24 h prior to transfection to achieve ∼75% confluency at the time of transfection. Plasmids were mixed in 200 μL Opti-MEM (Gibco, Grand Island, NY) with 1 mg/mL PEI (polyethylenimine; 1:5 DNA:PEI ratio) and allowed to incubate for 15 min at room temperature. 0.5 μg of all plasmids were used except for 1 μg Flag-LRRK2 R1441C. For experiments involving overexpression of PPM1M D440N (Figure 7), plasmids were mixed in 300 μL Opti-MEM (Gibco) with 1 mg/mL PEI (1:3 DNA:PEI ratio) and incubated for 30 min at room temperature. 0.75 μg of all plasmids were used except for 1.25 μg Flag-LRRK2 R1441G. Transfection mixture was added dropwise to attached cells and incubated for 24 h prior to immunoblotting analysis. See detailed protocol here https://dx.doi.org/10.17504/protocols.io.bawuifew.

#### siRNA phosphatase screen

Dharmacon mouse phosphatase siRNA library was purchased from Horizon Discovery (Cambridge, UK). This library does not contain all phosphatase genes; therefore a cherry-picked custom library of additional genes was also purchased (see Table S1) from Horizon Discovery. Low passage 3T3 Flp-In mouse fibroblasts were plated in 6-well plates at ∼30% confluency (200,000 cells/well) in 1.6 mL complete DMEM, 16–24 h prior to transfection. siRNA resuspension in Dharmafect 1 transfection reagent (Dharmacon, Lafayette, CO) was performed according to the manufacturer. In brief, siRNA was resuspended in 1x siRNA buffer (Dharmacon) to a final concentration of 20 μM. Each well was transfected with 25 nM siRNA and 4 μL Dharmafect 1 in a total of 400 μL Opti-MEM. Transfection mixture was added dropwise to each well. After 24 h, transfection media was replaced with fresh media, and cells were maintained for another 48 h. 100 nM of MLi-2 was then added to each well for 20 min prior to harvesting. In some control samples, 100 nM MLi-2 was added to cells for 2 h. Cells were harvested, lysed, and analyzed by immunoblotting. See detailed protocol here: https://dx.doi.org/10.17504/protocols.io.36wgqdxr5vk5/v1.

#### Pooled CRISPR knockout

Pooled CRISPR knockouts were generated by electroporation using guide RNAs designed and synthesized from Synthego. A detailed protocol can be found here: https://dx.doi.org/10.17504/protocols.io.bp2l6dqodvqe/v1. In brief, the top two ranked sgRNA per gene as determined by Synthego Knockout Design Tool (https://design.synthego.com) were pooled and combined with Cas9 (IDT) at a 6:1 sgRNA:Cas9 ratio to form an RNP complex. This RNP complex was electroporated into cells resuspended in Opti-MEM using a 0.2 cm cuvette and NEPA21 Type II electroporator. Cells were allowed to recover and expand for one week. Cells were then genotyped by extracting genomic DNA from cell pellets using QuikExtract and PCR amplification and sequencing of the targeted regions. Sequencing results were analyzed using Synthego ICE analysis (https://ice.synthego.com). Knockout was also validated by immunoblotting if endogenous protein levels were detectable (for PPM1H only, not PPM1M or PPM1J).

#### Isolation of PPM1M knockout MEFs

Wild-type, heterozygous, and homozygous PPM1M knockout MEFs were isolated from littermate matched mouse embryos at day E12.5 resulting from crosses between heterozygous PPM1M knockout and wild-type mice using the protocol described in https://doi.org/10.17504/protocols.io.eq2ly713qlx9/v1. Genotypes were verified via allelic sequencing and immunoblotting analysis. Cells were cultured in DMEM containing 10% (v/v) FBS, 2 mM L-glutamine, penicillin-streptomycin 100 U/mL, 1 mM sodium pyruvate, and 1x non-essential amino acid solution. Genotyping was performed by TransnetYX (https://www.transnetyx.com/) using the following probes: Ppm1m-1 WT and Ppm1m Tm2b as specified by the vendor.

#### Mouse tissue homogenization, cell lysis and immunoblotting analysis

Quantitative immunoblotting analysis from cultured cell lysates was performed as described in https://doi.org/10.17504/protocols.io.bsgrnbv6. Briefly, cell pellets were collected and lysed in lysis buffer (50 mM Tris–HCl pH 7.4, 150 nM NaCl, 1 mM EGTA, 2% glycerol, cOmplete EDTA-free protease inhibitor cocktail (Roche), PhosSTOP phosphatase inhibitor cocktail (Roche), 1 μg/mL microcystin-LR (Sigma), and 1% (v/v) Triton X-100). Lysates were clarified by centrifugation at 10,000 x g at 4°C for 10 min.

For mouse tissues, after dissection, tissues were homogenised using the Precellys Tissue Homogeniser in Tissue Grinding CKMix50-R tubes (P000922-LYSK0-A) with lysis buffer (50 mM Tris–HCl pH 7.4, 150 nM NaCl, 1 mM EGTA, 2% glycerol, cOmplete EDTA-free protease inhibitor cocktail (Roche), PhosSTOP phosphatase inhibitor cocktail (Roche), 1 μg/mL microcystin-LR (Sigma), and 1% (v/v) Triton X-100) at 6800 rpm for 3 × 20 s, with 30 s between each round of lysis. Following homogenisation, lysates were clarified by centrifugation at 10,000 x g at 4°C for 10 min.

Lysate protein concentration was determined by Bradford assay. Samples containing equal protein amounts were mixed with 5x SDS sample buffer (250 mM Tris-HCl, pH 6.8, 30% glycerol (v/v), 10% SDS (w/v), 0.1% bromophenol blue (w/v), 10% 2-mercaptoethanol (BME) (v/v)) and resolved on 4–20% precast gels (Bio-Rad) then transferred onto nitrocellulose membranes using the Transblot Turbo System (Bio-Rad). Membranes were blocked in 5% milk with TBST for 1 h and incubated with specific primary antibodies overnight at 4°C.

See key resources table for primary and secondary antibodies used and their dilutions. Primary antibodies were diluted in 3% BSA in Tris-buffered saline (200 mM Tris, 1.5M NaCl) with 0.1% Tween 20 (TBST) and detected using LI-COR IRdye labeled secondary antibodies (donkey anti-mouse 680/800, donkey anti-rabbit 680/800), diluted 1:10,000 in 5% milk in TBST. Membranes were scanned on the LICOR Odyssey DLx scanner. Images were saved as.tif files and analyzed using the gel scanning plugin in ImageJ.

#### Protein purification

His-SUMO-PPM1M, His-SUMO-Rab10 Q68L, and His-SUMO-Rab12 Q101L were purified after expression in E. coli BL21 (DE3 pLys). A detailed protocol can be found at https://doi.org/10.17504/protocols.io.81wgbnp9ygpk/v1. In brief, bacterial cells were grown at 37°C in Luria Broth and induced at A600 nm = 0.6 by the addition of 0.3 mM isopropyl-1-thio-β- d-galactopyranoside (IPTG) (Gold Biotechnology, St. Louis, MO) and harvested after growth for 18 h at 18°C. The cell pellets were resuspended in ice-cold lysis buffer (50 mM HEPES, pH 8.0, 10% (v/v) glycerol, 500 mM NaCl, 10 mM imidazole, 5 mM MgCl_2_, 0.2 mM TCEP, 20 μM GTP, and cOmplete EDTA-free protease inhibitor cocktail (Roche). The resuspended bacteria were lysed by one passage through an Emulsiflex-C5 apparatus (Avestin) at 10,000 lbs/in^2^ and centrifuged at 40,000 rpm for 45 min at 4°C in a Beckman Ti45 rotor.

Cleared lysate was filtered through a 0.2 μm filter (Nalgene) and passed over a HiTrap TALON crude 1 mL column (Cytiva). The column was washed with lysis buffer until absorbance values reached pre-lysate values. Protein was eluted with a gradient from 20 to 500 mM imidazole-containing lysis buffer. Peak fractions were analyzed by 4–20% SDS-PAGE to locate protein. If cleaving off His-SUMO tag (for Rab10 and Rab12), the eluate was cleaved overnight using homemade SUMO protease while dialyzing into a buffer containing 50 mM HEPES pH 8, 5% (v/v) glycerol, 150 mM NaCl, 5 mM MgCl_2_, 0.2 mM TCEP, and 20 μM GTP. The cleaved product was then further purified by gel filtration on Superdex 200 16/60 120 mL or Superdex 200 10/300 24 mL size exclusion columns (Cytiva) using a buffer containing 50 mM HEPES pH 8, 5% (v/v) glycerol, 150 mM NaCl, 5 mM MgCl_2_, 0.2 mM TCEP, and 20 μM GTP. Fractions were resolved on a 4–20% Mini-PROTEAN TGX Gel (Bio-Rad) and visualized using InstantBlue Coomassie Stain (Abcam, Waltham, MA).

#### *In vitro* phosphatase assay with immunoblotting analysis

A detailed protocol can be found at https://doi.org/10.17504/protocols.io.5jyl8d4j7g2w/v1. Briefly, untagged Rab10 Q68L or untagged Rab12 Q101L was incubated with His-MST3 kinase in a reaction buffer (50 mM HEPES pH 8, 100 mM NaCl, 5 mM MgCl_2_, 2 mM ATP, 100 μM GTP, 0.5 mM TCEP, 10% glycerol) at 4°C overnight to phosphorylate Rab10/Rab12. Next, His-MST3 kinase was removed by passing the sample through a 1-mL syringe column containing 100 μL (50%) Ni-NTA slurry; the flow through containing phosphorylated Rab10 or Rab12 was collected. For phosphatase assays, 1.5 μM pRab10 or pRab12 was incubated with 50 or 100nM His-SUMO-PPM1M at 30°C for various times. A master mix reaction was made using 15 μL total reaction volume per time point. At the indicated time point, 15 μL was removed from the reaction tube and reaction aliquots were stopped by addition of 5 μL SDS-PAGE sample buffer. Samples were then analyzed by immunoblotting to detect dephosphorylation of Rab10 or Rab12 using anti-pRab10 antibody or anti-pRab12 antibody as above.

#### Chromatography of HEK293 cytosol

Crude membrane fractionation was performed according to https://doi.org/10.17504/protocols.io.yxmvmnb99g3p/v1. Briefly, 70% confluent HEK293 cells were transfected in 10 cm dishes with 5 μg HA-PPM1M plasmid. 24 h after transfection, 3 × 10 cm dishes of cells were washed 2x with ice-cold PBS, pooled, and swollen in 400 μL of hypotonic buffer (10 mM HEPES pH 7.4 with protease inhibitors). After 20 min, 100 μL of 5X resuspension buffer was added to achieve a final concentration of 1X resuspension buffer (50 mM HEPES pH 7.4, 150 mM NaCl, 5 mM MgCl_2_, 1X cOmplete protease inhibitor cocktail (Roche), 1X phosphatase PhosStop inhibitor (Roche)). The suspension was passed 40 times through a 27G needle. Lysate was spun at 1,000 X g for 5 min at 4°C to pellet nuclei. The postnuclear supernatant was spun at 55,000 RPM for 20 min at 4°C in a tabletop ultracentrifuge in a TLA100.2 rotor; the resulting supernatant (∼500 μL) was collected as the cytosol fraction. The supernatant was then applied onto a 24 mL Superdex 10/300 column (Cytiva) and fractions subjected to immunoblot analysis to determine PPM1M elution. Corresponding mass (kDa) was determined by comparison with calibration standards (Bio-Rad).

#### *In vitro* pRab8A phosphatase assays with Phos-tag gel analysis

Bacterial expression and purification of 6xHIS-SUMO-PPM1M (WT, H127D, D440N), PPM1H (WT, D288A) and large scale preparation of phosphorylated GTPγS-bound Rab8A Thr72 were performed as previously described (https://doi.org/10.17504/protocols.io.bu7wnzpe and https://doi.org/10.17504/protocols.io.butinwke). For *in vitro* phosphatase assays, 2.5 μg phosphorylated, GTPγS-bound Rab8A was incubated with varying amounts of recombinant wild-type PPM1M or PPM1H or their respective variants in a reaction buffer containing 40 mM HEPES (pH 7.0) and 10 mM MgCl_2_. GTPγS-bound pRab8A was in 20 mM MES pH 5.3, 0.1 M NaCl, 10% (by vol) glycerol, 0.03% (by vol) Brij 35, 14 mM 2-mercaptoethanol, 2 mM MgCl_2_, 1 mM GTPγS while PPM1M/H proteins were in a buffer containing 50 mM Tris/HCl pH 7.5, 150 mM NaCl, 2 mM MnCl_2_, 0.5 mM TCEP buffer. Following a 30-min incubation, reactions were terminated by adding 6uL of 4x lithium dodecyl sulfate (LDS) (106 mM Tris HCl, 141 mM Tris Base, 2% (w/v) LDS, 10% (v/v) glycerol, 0.51 mM EDTA, 0.22 mM SERVA Blue G250, 0.175 mM Phenol Red, pH 8.5) supplemented with 5% (v/v) 2-mercaptoethanol. Samples were analysed via Phos-Tag gel electrophoresis as described by Ito et al.^32^ Post-electrophoresis, the gel was stained with Instant Blue Coomassie (Abcam) for subsequent analysis.

#### Mouse brain processing

Ppm1m^−/−^ (3-month-old) mice and their littermate wild-type controls (both of mixed gender) were fixed by transcardial perfusion using 4% paraformaldehyde (PFA) in PBS as described in https://doi.org/10.17504/protocols.io.bnwimfce. Whole brain tissue was extracted, post-fixed in 4% PFA for 24 h and then immersed in 30% (w/v) sucrose in PBS until the tissue settled to the bottom of the tube (∼48 h). The brains were harvested in Dundee and sent to Stanford with identities blinded until analysis was completed. Prior to cryosectioning, brains were embedded in cubed-shaped plastic blocks with OCT (BioTek, USA) and stored at −80°C. OCT blocks were allowed to reach −20°C for ease of sectioning. The brains were oriented to cut coronal sections on a cryotome (Leica CM3050S, Germany) at 25 μm thickness and positioned onto SuperFrost plus tissue slides (Thermo Fisher, USA).

#### Immunohistochemical staining

Mouse brain striatum was subjected to immunostaining following a previously established protocol (https://dx.doi.org/10.17504/protocols.io.bnwimfce). Frozen slides were thawed at room temperature for 15 min and then gently washed twice with PBS for 5 min each. Antigen retrieval was achieved by incubating the slides in 10 mM sodium citrate buffer pH 6.0, preheated to 95°C, for 15 min. Sections were permeabilized with 0.1% Triton X-100 in PBS at room temperature for 15 min, followed by blocking with PBS containing 2% FBS and 1% BSA for 2 h at room temperature. Primary antibodies were applied overnight at 4°C, and sections were then exposed to secondary antibodies at room temperature for 2 h. Secondary antibodies used were donkey highly cross-absorbed H + L antibodies conjugated to Alexa 488 and Alexa 568 diluted at 1:2000. Nuclei were counterstained with 0.1 μg/mL DAPI (Sigma). Finally, stained tissues were mounted with Fluoromount G and covered with a glass coverslip. All antibody dilutions for tissue staining contained 1% DMSO to facilitate antibody penetration.

#### Microscope image acquisition

All images were obtained using a Zeiss LSM 900 confocal microscope (Axio Observer Z1/7) coupled with an Axiocam 705 camera and immersion objective (Plan-Apochromat 63×/1.4 Oil DIC M27). The images were acquired using ZEN 3.4 (blue edition) software, and visualizations and analyses were performed using ImageJ Fiji.

#### Genetic dataset analysis for PPM1M variants

For the Vienna samples, Whole-Exome Sequencing was performed at the Munich Genome Analysis Competence Center (Klinikum rechts der Isar; Technische Universität München, Germany).^33^ Details of GP2 whole genome sequencing and bioinformatic pipelines have been previously reported.^34^ Briefly, samples were sequenced to an average of 30x coverage with 150 bp paired-end reads, following Illumina’s TruSeq PCR-free library preparation protocol. Sequencing reads were aligned to the human reference genome (GRCh38 build) using the functional equivalence pipeline,^35^ and variant calling was performed as described.^36,37^ Samples were retained for downstream analyses after passing quality control based on the quality metrics defined by the Accelerating Medicines Partnership Parkinson’s Disease program (AMP-PD; https://amp-pd.org).^38^ For the ROPAD study, DNA was extracted from dried blood spots on filter cards (CentoCard^®^) using standard, spin column-based methods, followed by sequencing on the HiSeqX platform (Illumina) to yield an average coverage depth 41x. Variant annotation was performed as described.^4^

### QUANTIFICATION AND STATISTICAL ANALYSIS

All statistical details of experiments can be found in the figure legends, including the exact value of n, what n represents, definition of center, and dispersion and precision, and the statistical tests used.

### ADDITIONAL RESOURCES

GP2’s Data Release 10 (Zenodo Data: https://doi.org/10.5281/zenodo.7904831) was used to screen for *PPM1M* D440N carriers.

## Supplementary Material

1

2

Supplemental information can be found online at https://doi.org/10.1016/j.celrep.2025.116031.

## Figures and Tables

**Table:** KEY RESOURCES TABLE

REAGENT or RESOURCE	SOURCE	IDENTIFIER
Antibodies		

anti-GAPDH (mouse monoclonal), 1:5000	Santa Cruz Biotechnology	Cat#sc-32233; RRID: AB_627679
anti-PPM1H (rabbit monoclonal), 1:2000	Abcam	Cat#ab303536; RRID: AB_2941812
anti-alpha tubulin (mouse monoclonal), 1:4000	Santa Cruz Biotechnology	Cat#sc-32293; RRID: AB_628412
anti-HA (rabbit polyclonal), 1:1000	Sigma	Cat#H6908; RRID: AB_260070
anti-HA (mouse monoclonal), 1:1000	Sigma	Cat#H9658; RRID: AB_260092
anti-HA (rat monoclonal), 1:1000	Sigma	Cat#11867423001; RRID: AB_390918
anti-LRRK2 (mouse monoclonal), 1:1000	Antibodies Incorporated/NeuroMab	Cat#N241A/34; RRID: AB_10675136
anti-LRRK2 pS935 (rabbit monoclonal), 1:1000	MRC PPU Reagents and Services, U. Dundee and/or Abcam Inc.	Cat#ab133450; RRID: AB_2732035
anti-Rab10 pThr73 (rabbit monoclonal), 1:1000	Abcam Inc.	Cat#ab230261; RRID: AB_2811274
anti-Rab10 (mouse monoclonal), 1:1000	Abcam Inc.	Cat#ab104859; RRID: AB_10711207
anti-Rab12 pSer106 (rabbit monoclonal), 1:1000	Abcam Inc.	Cat#ab256487; RRID: AB_2884880
anti-Rab12 (mouse monoclonal), 1:500	Santa Cruz Biotechnology	Cat#sc-515613; RRID: AB_3101762
IRDye 800CW Donkey anti-Rabbit IgG, 1:10,000	LI-COR	Cat#926–32213; RRID: AB_621848
IRDye 680RD Donkey anti-Rabbit IgG, 1:10,000	LI-COR	Cat#926–68073; RRID: AB_10954442
IRDye 800CW Donkey anti-Mouse IgG, 1:10,000	LI-COR	Cat#926–32212; RRID: AB_621847
IRDye 680RD Donkey anti-Mouse IgG, 1:10,000	LI-COR	Cat#926–68072; RRID: AB_10953628
anti-Choline Acetyltransferase (goat polyclonal), 1:200	Sigma	Cat#208371; RRID:AB_2079751
anti-Adenylate cyclase III (rabbit polyclonal), 1:10,000	EnCOR	Cat#RPCA-ACIII; RRID:AB_2572219
H+L Donkey anti-Goat AF 488, 1:2000	Life Technologies	Cat#A11055; RRID:AB_2534102
H+L Donkey anti-Rabbit AF 568, 1:2000	Life Technologies	Cat#A10042; RRID:AB_2534017
H+L Donkey anti-guinea pig Alexa 647, 1:2000	Jackson ImmunoResearch	Cat#706–605-148; RRID: AB_2340476
anti-Parvalbumin (guinea pig polyclonal), 1:1000	Synaptic system	Cat#195004; RRID: AB_2156476

Bacterial and virus strains		

MAX Efficiency^™^ DH5α Competent Cells	Invitrogen	Cat#18258012
BL21(DE3)pLysS Competent Cells	Novagen	Cat#69451–3

Chemicals, peptides, and recombinant proteins		

MLi-2	MRC PPU Reagents and Services, U. Dundee	CAS No.: 1627091–47-7
Instant Blue Coomassie	Abcam	Cat#ab119211
Bio-Rad Protein Assay Dye Reagent Concentrate	Bio-Rad	Cat#5000006
Dharmafect 1	Dharmacon	Cat#T-2001
DMEM (high glucose)	Cytiva	Cat#SH30243.02
Fetal bovine serum	Sigma	Cat#F0926
Penicillin-Streptomycin	Sigma	Cat#P4333
Opti-MEM	Gibco	Cat#31985088
PEI, 25 kDa	Polysciences	Cat#23966
cOmplete EDTA-free protease inhibitor cocktail	Roche	Cat#11873580001
PhosSTOP phosphatase inhibitor cocktail	Roche	Cat#4906837001
Microcystin-LR	Sigma	Cat#475815-M
Mouse Rab12 siRNA (OnTarget, SMARTpool)	Dharmacon	Cat#L-040865–01
Non-targeting siRNA (OnTarget, SMARTpool)	Dharmacon	Cat#D-001810–10
Mouse phosphatase siRNA library (OnTarget, SMARTpool)	Dharmacon	Cat#G-113705, see Table S1 for gene list
Cherry-picked mouse phosphatase siRNA library (OnTarget, SMARTpool)	Dharmacon	Custom, see Table S1 for gene list
GoTaq Green Master Mix	Promega	Cat#M7122

Critical commercial assays		

4–20% Criterion TGX Precast Midi Protein Gel, 26 well, 15 μL	Bio-Rad	Cat#5671095
Trans-Blot Turbo RTA Midi 0.2 μm Nitrocellulose Transfer Kit	Bio-Rad	Cat#1704271
HiTrap TALON crude 1 mL column	Cytiva	Cat#28953766
Econospin column	Epoch Lifesciences	Cat#1920–050/250
Electroporation Cuvettes, 0.2 cm gap	Bio-Rad	Cat#1652086

Deposited data		

PPM1M, a LRRK2-counteracting, phosphoRab12-preferring phosphatase with potential link to Parkinson’s disease	Zenodo	https://doi.org/10.5281/zenodo.14911978
PPM1M, a LRRK2-counteracting, phosphoRab12-preferring phosphatase with potential link to Parkinson’s disease	Zenodo	https://doi.org/10.5281/zenodo.15499979

Experimental models: Cell lines		

HEK293T (human)	ATCC	CRL-3216; RRID: CVCL_0063
A549 (human)	ATCC	CCL-185; RRID: CVCL_0023
PPM1H knockout A549 (human)	MRC PPU Reagents and Services, U. Dundee	PMIID: 31663853
Mouse Embryonic Fibroblasts (mouse)	MRC PPU Reagents and Services, U. Dundee	RRID: CVCL_E7DI
PPM1M knockout Mouse Embryonic Fibroblasts (mouse)	MRC PPU Reagents and Services, U. Dundee	RRID: CVCL_E7DI
3T3 Flp In (mouse)	Invitrogen	R76107; RRID: CVCL_U422
Experimental models: Organisms/strains		
PPM1M knockout mouse	MRC Harwell	MGI ID: MGI:5638564
Recombinant DNA		
pET15b His-MST3	MRC PPU Reagents and Services, U. Dundee	DU62980
pCMV5D HA-PPM1H	MRC PPU Reagents and Services, U. Dundee	DU62789
pCMV5D HA-PPM1H H153D	MRC PPU Reagents and Services, U. Dundee	DU62928
pCMV5D HA-PPM1H D288A	MRC PPU Reagents and Services, U. Dundee	DU62985
pCMV5D HA-PPM1M	MRC PPU Reagents and Services, U. Dundee	DU68124
pCMV5D HA-PPM1M H127D	MRC PPU Reagents and Services, U. Dundee	DU68165
pCMV5D HA-PPM1M D235A	MRC PPU Reagents and Services, U. Dundee	DU68164
pCMV5D HA-PPM1M D440N	MRC PPU Reagents and Services, U. Dundee	DU72159
pCMV5D HA-PPM1H_M flap	Addgene	RRID: Addgene_236718
pCMV5D HA-PPM1M_H flap	Addgene	RRID: Addgene_236719
Flag-LRRK2 R1441C	MRC PPU Reagents and Services, U. Dundee	DU13078
Flag-LRRK2 R1441G	MRC PPU Reagents and Services, U. Dundee	DU26477
His-SUMO-PPM1M	MRC PPU Reagents and Services, U. Dundee	DU68141
His-SUMO-PPM1M H127D	MRC PPU Reagents and Services, U. Dundee	DU68200
His-SUMO-PPM1M D440N	MRC PPU Reagents and Services, U. Dundee	DU72158
His-SUMO-PPM1H	MRC PPU Reagents and Services, U. Dundee	DU62835
His-SUMO-PPM1H D288A	MRC PPU Reagents and Services, U. Dundee	DU68087
His-Thrombin-Rab8A (1–181)	MRC PPU Reagents and Services, U. Dundee	DU68198
His-SUMO-Rab10 Q68L	Addgene	RRID: Addgene_236720
His-SUMO-Rab12 Q101L	Addgene	RRID:Addgene_208371

Software and algorithms		

Alphafold Server	Alphafold 3	RRID: SCR_025885
ChimeraX	ChimeraX	PMID: 32881101; RRID: SCR_015872
ImageJ	ImageJ version 2.14	RRID: SCR_003070
Graphpad Prism	Prism 10 version 10.2.3	RRID: SCR_002798
ZEN	Zeiss ZEN Microscopy Software	RRID: SCR_013672
Synthego ICE	Inference of CRISPR Edits	RRID: SCR_024508

## Highlights

- PPM1M is an LRRK2-counteracting, phosphoRab12-preferring phosphatase
- *Ppm1m* knockout triggers cilia loss in mouse striatal cholinergic and parvalbumin interneurons
- A rare, catalytically inactive *PPM1M* D440N variant occurs ∼6-fold more often in Parkinson’s cases
- PPM1M provides a new therapeutic target for the possible benefit of people with Parkinson’s
