# Supplementary material for: PPM1M, an LRRK2-counteracting, phosphoRab12-preferring phosphatase with a potential link to Parkinson’s disease
Source: Cell Rep. Author manuscript; Available in PMC 2025 Sep 25. (PMC12462683; doi:10.1016/j.celrep.2025.116031)
Supplement: 1 [file NIHMS2107258-supplement-1.pdf]

Cell Reports, Volume *44*

Supplemental information

PPM1M, an LRRK2-counteracting, phosphoRab12-preferring phosphatase with a potential link to Parkinson's disease

Claire Y. Chiang, Neringa Pratuseviciute, Yu-En Lin, Ayan Adhikari, Wondwossen M. Yeshaw, Chloe Flitton, Pemba L. Sherpa, Francesca Tonelli, Irena Rektorova, Timothy Lynch, Joanna Siuda, Monika Rudzińska-Bar, Oleksandr Pulyk, Peter Bauer, Christian Beetz, Dennis W. Dickson, Owen A. Ross, Zbigniew K. Wszolek, Global Parkinson's Genetics Program (GP2), Zih-Hua Fang, Christine Klein, Alexander Zimprich, Dario R. Alessi, Esther M. Sammler, and Suzanne R. Pfeffer

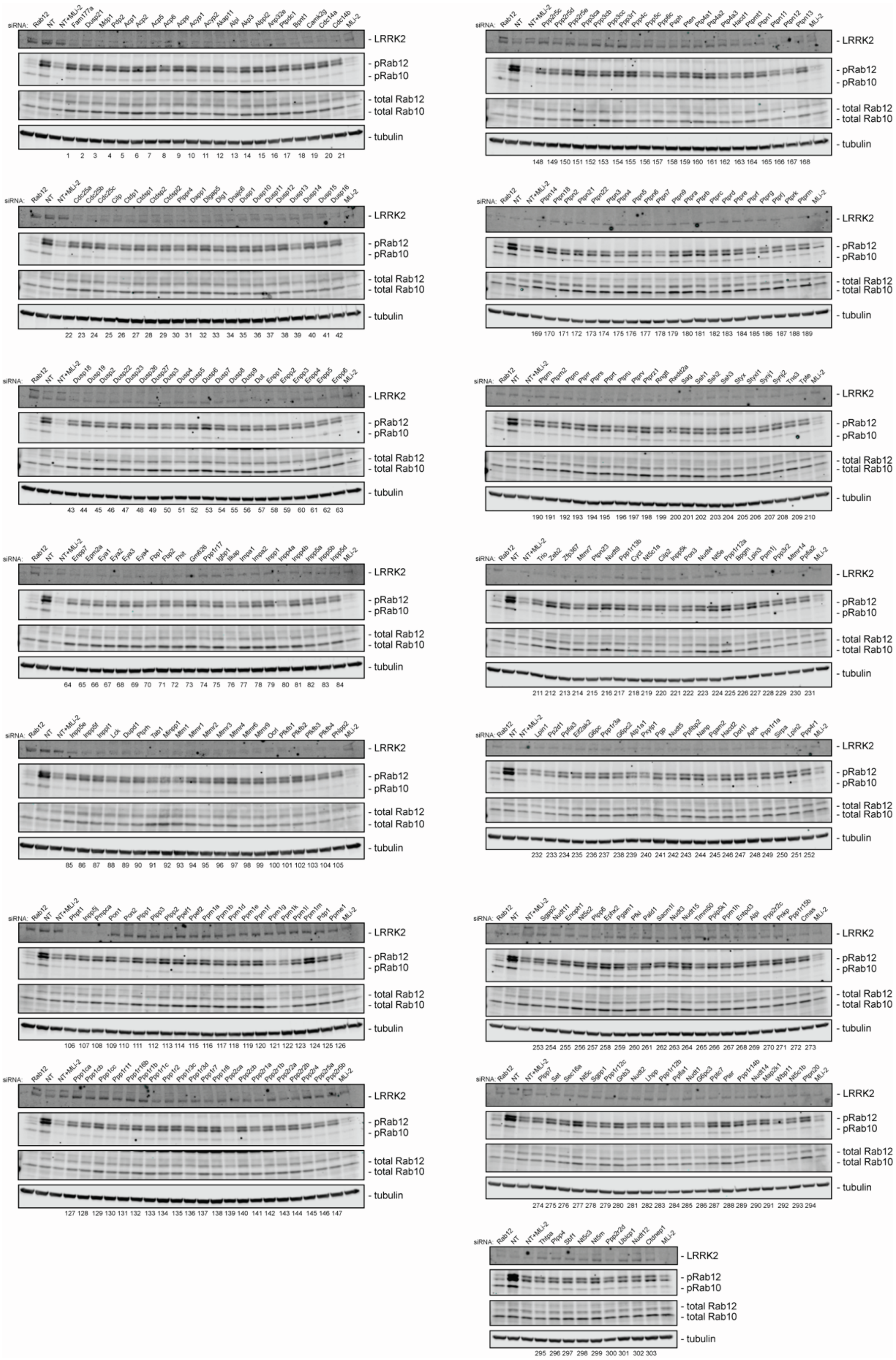

Figure S1. Immunoblots used for screen quantitation. Each lane represents a unique siRNA from the library or Rab12 siRNA, non-targeting siRNA, or non-targeting siRNA with MLi-2. siRNAs were identified by ID number only (labeled below each blot) during screen and analysis. A list of all genes and guides is included in Supplemental Table 2.

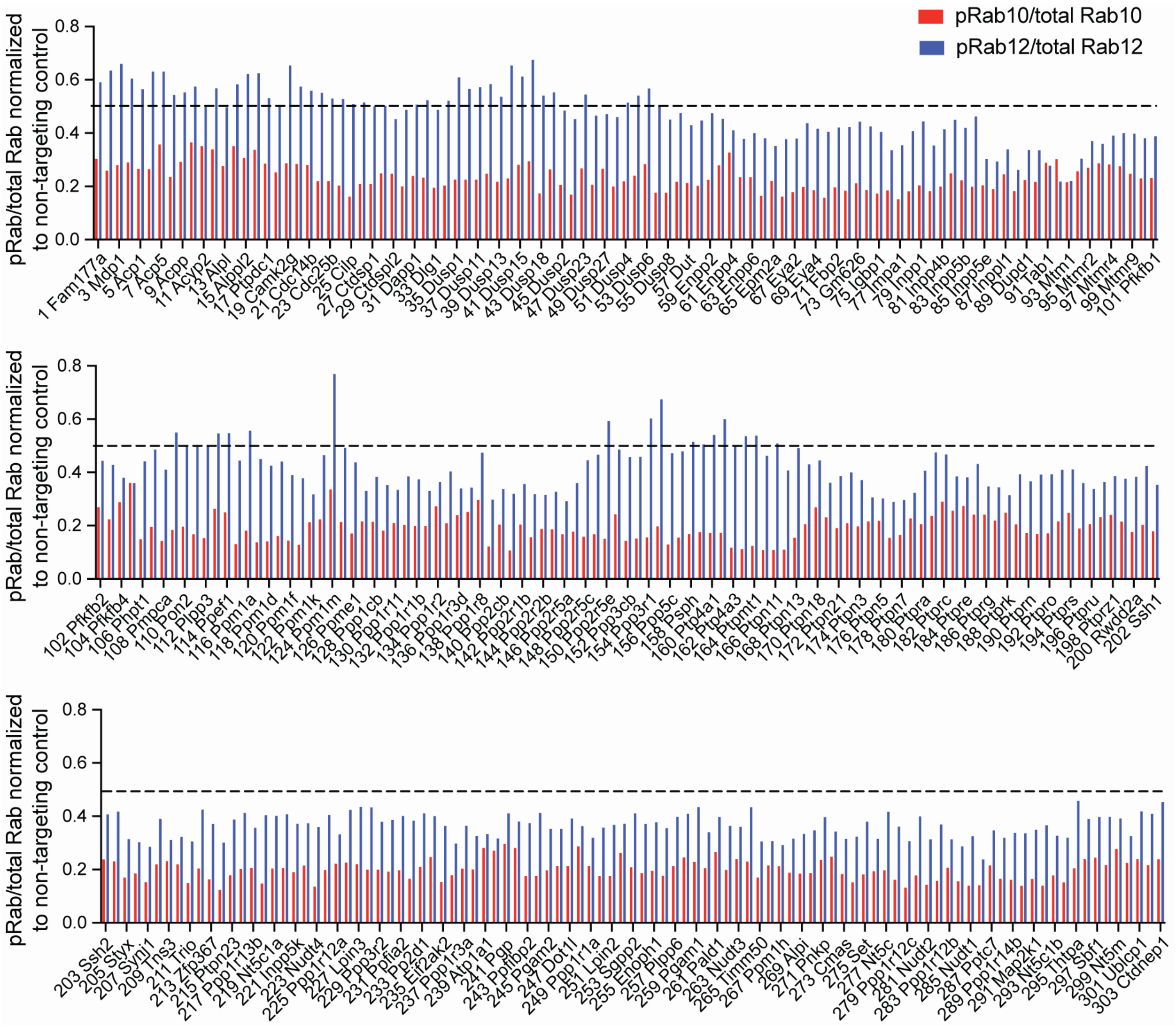

Figure S2. Quantitative analysis of the phosphatome-wide screen. Quantitation of pRab12/total Rab12 and pRab10/total Rab10 from gels in Supplemental Figure 1, normalized to non-targeting (NT) control. Values are the average of two independent analyses of the same data.

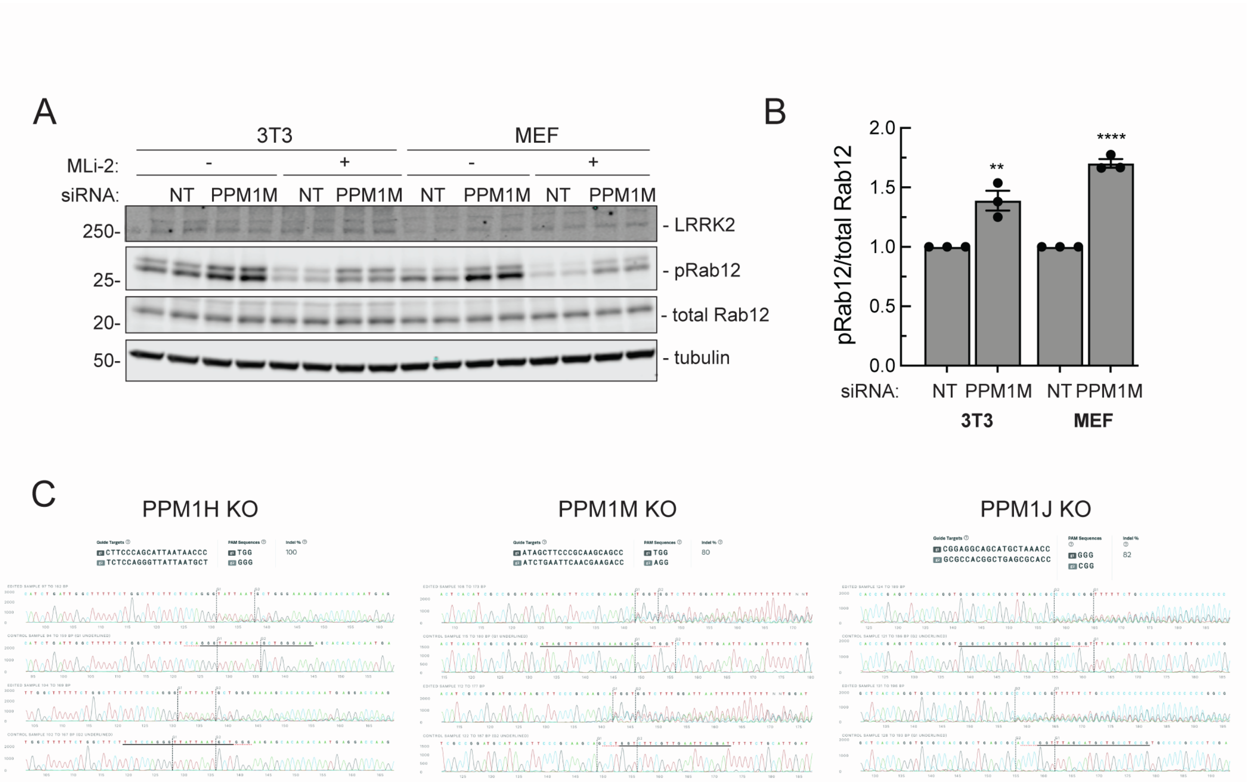

Figure S3. PPM1M knockdown in 3T3 and MEF cells increases pRab12 levels. (A) Immunoblot analysis of 3T3 and MEF cells treated with non-targeting (NT) or PPM1M siRNA for 72h, followed by 200 nM MLi-2 for 20 minutes as indicated. **(B)** Quantitation of pRab12 levels from immunoblots in (A) normalized to respective NT controls. Error bars indicate SEM from three independent experiments carried out in duplicate. Statistical significance determined by student's T-test, respective to NT. **p=0.0093 for 3T3, ****p<0.0001 for MEF. **(C)** Genotyping results of pooled CRISPR knockouts for *Ppm1h*, *Ppm1m*, and *Ppm1j* MEFs by Synthego ICE software. For each cell line, guide sequences, indel frequency (%), and sequencing traces for edited and control samples for each guide are shown.

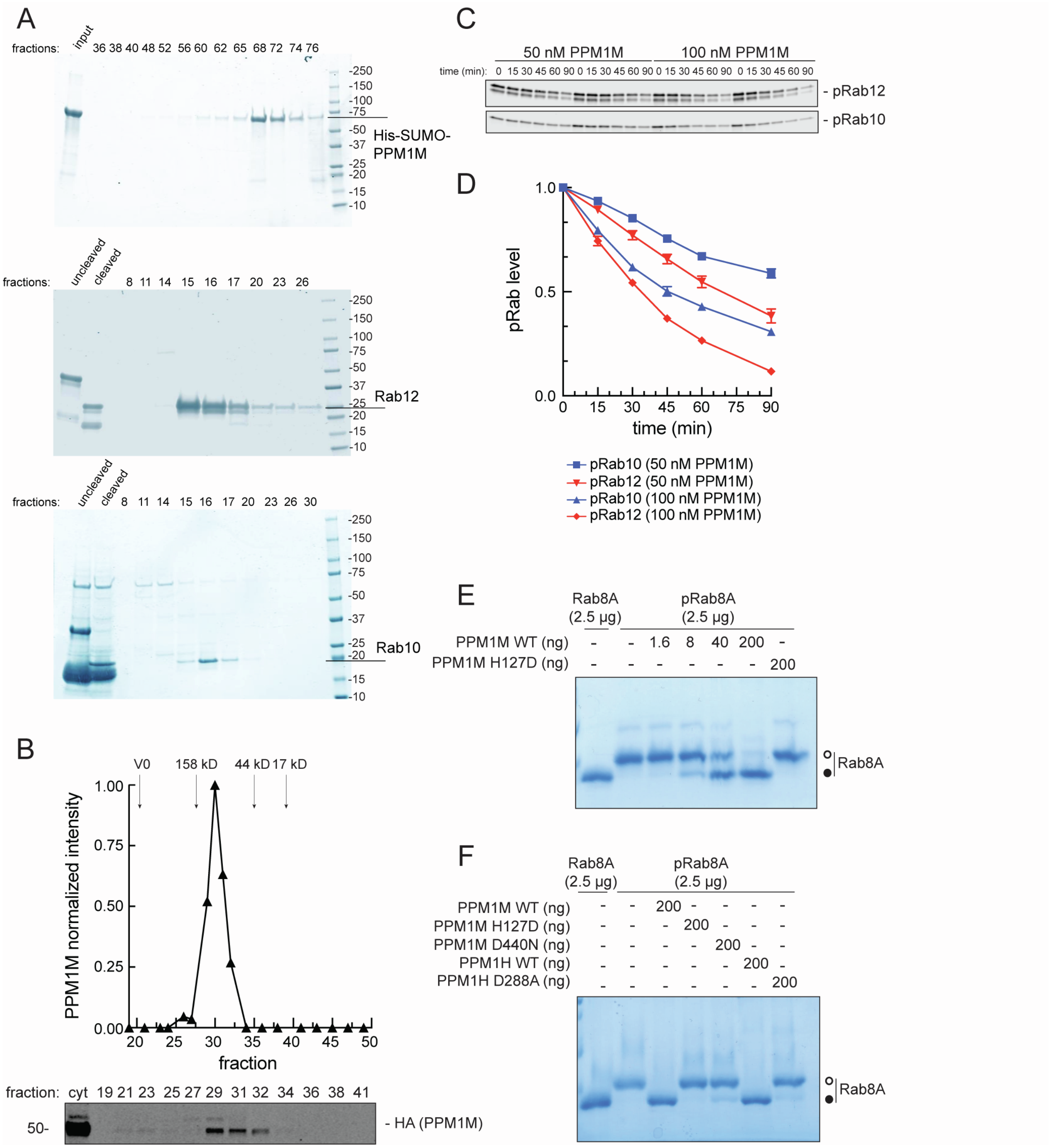

Figure S4. PPM1M prefers phosphoRab12 over phosphoRab10 *in vitro*. (A) SDS-PAGE elution of His-SUMO-PPM1M after Superdex 200 16/60 (top row) and SUMO-tag cleaved (His-SUMO-)Rab10 (middle panel) or (His-SUMO-)Rab12 (bottom panel) after Superdex 200 10/300. Fraction 72 was used for His-SUMO-PPM1M. Mass is shown at right in kDa. **(B)** PPM1M chromatographs as a dimer in cytosol. HEK293 cytosol overexpressing HA-PPM1M was resolved on Superdex 200 10/300. Immunoblot of fractions and their quantitation is shown. **(C)** Immunoblot of pRab12 and pRab10 levels from *in vitro* biochemical reactions using 1.5µM pRab12 or pRab10 substrate and 50 or 100nM PPM1M enzyme, as indicated. **(D)** Quantitation of pRab12 and pRab10 levels from immunoblots in (A), normalized to 1.0 for 0 min. Error bars represent SEM from five independent experiments. **(E)** Phos-tag gel analysis of pRab8A dephosphorylation after *in vitro* reactions containing 2.5 µg pRab8A and indicated amounts of PPM1M. **(F)** Phos-tag gel analysis of pRab8A dephosphorylation after biochemical reactions containing 2.5 µg pRab8A and 200 ng of the indicated phosphatases.

Table S2. Overview of cohorts interrogated for *PPM1M* p.D440N carrier status. Included is total number of individuals per study, number of disease and control subjects, number of individuals carrying the *PPM1M* D440N variant in the heterozygous state with and without PD, and minor allele frequency (MAF). Abbreviations: DLB, dementia with Lewy Bodies; GP2, Global Parkinson´s Genetics Program, prodr: prodromal case with either REM sleep behavior disorder or hyposmia; PSP, Progressive Supranuclear Palsy, YOPD, young onset PD.

Study	N	Disease / control cohort (if applicable)	*PPM1M* D440N PD+PD-Parkinsonism	*PPM1M* D440N non-PD	*PPM1M* D440N Allele Frequency cases/controls
Hop et.al.[29]	71959	2184 (familial PD cases) / 69775 controls	3 PD	3	6.87E-04/2.151E-05
Austrian PD cases	382	382 (familial and YOPD cases)	1 PD	n.a.	1.31E-03
Mayo Clinic cases	700	700 (neuropathological Specimens)	1 DLB	n.a.	7.14E-04
Czechia PD cases	33	33	0	n.a.	0
Ireland PD cases	672	672	0	n.a.	0
Poland PD cases	725	725	1 PD	n.a.	6.90E-04
Ukraine PD cases	139	139	1 PD	n.a.	3.60E-03
gnomAD (vs.4.1)	806916	806916 (general population)	N/A	66	4.13E-05
Centogene	192000	10000 PD cases / 182000 non-PD cases	0	13	0/ 3.57E-05
GP2	23413	13332 cases/ 10081 controls	4 (2PD+1PSP+ 1prodr)	0	2E-04/ 0
